# Water deficit alters differentially metabolic pathways affecting important flavor and quality traits in grape berries of Cabernet Sauvignon and Chardonnay

**DOI:** 10.1186/1471-2164-10-212

**Published:** 2009-05-08

**Authors:** Laurent G Deluc, David R Quilici, Alain Decendit, Jérôme Grimplet, Matthew D Wheatley, Karen A Schlauch, Jean-Michel Mérillon, John C Cushman, Grant R Cramer

**Affiliations:** 1 Department of Biochemistry and Molecular Biology, Mail Stop 200, University of Nevada, Reno, Nevada 89557, USA; 2 Groupe d'Études des Substances Végétales à Activité Biologique, EA 3675, Institut des Sciences de la Vigne et du Vin, Université *Victor Segalen *Bordeaux 2, UFR Sciences Pharmaceutiques, 146 rue Léo Saignat, 33076 Bordeaux Cedex, France

## Abstract

**Background:**

Water deficit has significant effects on grape berry composition resulting in improved wine quality by the enhancement of color, flavors, or aromas. While some pathways or enzymes affected by water deficit have been identified, little is known about the global effects of water deficit on grape berry metabolism.

**Results:**

The effects of long-term, seasonal water deficit on berries of Cabernet Sauvignon, a red-wine grape, and Chardonnay, a white-wine grape were analyzed by integrated transcript and metabolite profiling. Over the course of berry development, the steady-state transcript abundance of approximately 6,000 Unigenes differed significantly between the cultivars and the irrigation treatments. Water deficit most affected the phenylpropanoid, ABA, isoprenoid, carotenoid, amino acid and fatty acid metabolic pathways. Targeted metabolites were profiled to confirm putative changes in specific metabolic pathways. Water deficit activated the expression of numerous transcripts associated with glutamate and proline biosynthesis and some committed steps of the phenylpropanoid pathway that increased anthocyanin concentrations in Cabernet Sauvignon. In Chardonnay, water deficit activated parts of the phenylpropanoid, energy, carotenoid and isoprenoid metabolic pathways that contribute to increased concentrations of antheraxanthin, flavonols and aroma volatiles. Water deficit affected the ABA metabolic pathway in both cultivars. Berry ABA concentrations were highly correlated with 9-cis-epoxycarotenoid dioxygenase (*NCED1*) transcript abundance, whereas the mRNA expression of other *NCED *genes and ABA catabolic and glycosylation processes were largely unaffected. Water deficit nearly doubled ABA concentrations within berries of Cabernet Sauvignon, whereas it decreased ABA in Chardonnay at véraison and shortly thereafter.

**Conclusion:**

The metabolic responses of grapes to water deficit varied with the cultivar and fruit pigmentation. Chardonnay berries, which lack any significant anthocyanin content, exhibited increased photoprotection mechanisms under water deficit conditions. Water deficit increased ABA, proline, sugar and anthocyanin concentrations in Cabernet Sauvignon, but not Chardonnay berries, consistent with the hypothesis that ABA enhanced accumulation of these compounds. Water deficit increased the transcript abundance of lipoxygenase and hydroperoxide lyase in fatty metabolism, a pathway known to affect berry and wine aromas. These changes in metabolism have important impacts on berry flavor and quality characteristics. Several of these metabolites are known to contribute to increased human-health benefits.

## Background

*Vitis vinifera *L. has relatively high drought tolerance [1,2]. Once established in a deep soil with adequate water retention characteristics, grapevines produce root systems several meters deep enabling the vines to survive severe water deficits. Grape sensitivity to water deficit depends on the timing of the application being particularly more sensitive during anthesis and just after anthesis [3,4].

Regulated-deficit irrigation has been used to improve berry and wine quality [5,6]. For instance, application of water deficit early in the season before véraison resulted in greater concentrations of anthocyanins and phenolics [7,8]. Color differences were the result of increased anthocyanin synthesis caused by water deficit applied either early or late in the season [7,9]. Indeed, water deficit treatment typically increases the skin to pulp ratio compared to well-watered vines [10,6], increasing the amount of skin tannins and anthocyanins. However, it does not appear to affect the quantity or polymerization of seed tannins [11,6]. Application of water deficit after véraison decreases berry weight to a lesser extent [4], while still increasing substantially phenolic compounds such as anthocyanins [7]. In addition, the rates of accumulation of flavonoids as well as the degree of tannin polymerization may be increased [2]. In contrast, excessive and prolonged irrigation can reduce grape quality and yield due to late season vine growth [12]. Prolonged irrigation can delay or reduce sugar accumulation and increase titratable acidity. In addition, prolonged irrigation can reduce anthocyanin content, in part due to continuous and excessive shoot growth that increases the shading of berry clusters [13].

Whereas physiological and biochemical data are numerous regarding the effect of water deficit, little is known about gene expression in grape berries exposed to water deficit. One global approach, using the Affymetrix^® ^*Vitis vinifera *(Grape) Genome Array V. 1.0, at the tissue level indicated that water deficit affected the mRNA abundance of 13% of genes at grape maturity within the three tissues of the berry (skin, pulp and seeds), with the greatest changes located in the pulp and skin [14]. While the function of many of the genes differentially expressed within the seed and pulp remain to be elucidated, genes over-represented in the skin were clearly associated with phenylpropanoid metabolism, ethylene, pathogenesis-related responses, energy metabolism and stress responses.

Recently, water deficit was shown to accelerate anthocyanin biosynthesis, along with associated changes in transcript abundance of genes in the anthocyanin biosynthesis pathway in the grape berry skin [9,15]. These authors suggested that both ABA and sugar signaling might have accelerated anthocyanin development. Indeed, additions of ABA and rhamnose to grape berry skins have induced anthocyanin biosynthesis in a synergistic manner [16].

Water deficit is known to increase ABA concentrations in the xylem sap and leaves of grapevine [17-19]. Under normal conditions (no water deficit), ABA concentrations in berries peak near véraison [20], when berries begin to soften, and sugars and anthocyanins begin to accumulate. To the best of our knowledge, only one report has described the effect of water deficit on ABA concentrations in grape berries [18]; water-deficits applied after véraison significantly increased ABA concentrations in Chardonnay berries in one water-deficit treatment, but not in another water-deficit treatment that occurred in a later developmental stage.

In this study, we tested the hypothesis that a long-term, seasonal water deficit induces ABA concentrations in the berries of red-wine grape cultivar, Cabernet Sauvignon, influencing berry phenylpropanoid and sugar metabolism after véraison. Likewise, we expected that a long-term, seasonal water deficit would cause different metabolic responses in Chardonnay, a white-wine grape cultivar that lacks anthocyanin biosynthesis. We show that there are significant differences in the response to water deficit between the two cultivars. Not only were there differences in the effects of water deficit on phenylpropanoid metabolism, but also there were different effects of water deficit on ABA, isoprenoid, carotenoid, amino acid and fatty acid metabolism.

## Results

### Physiological responses to water deficit

**Figure 1:**
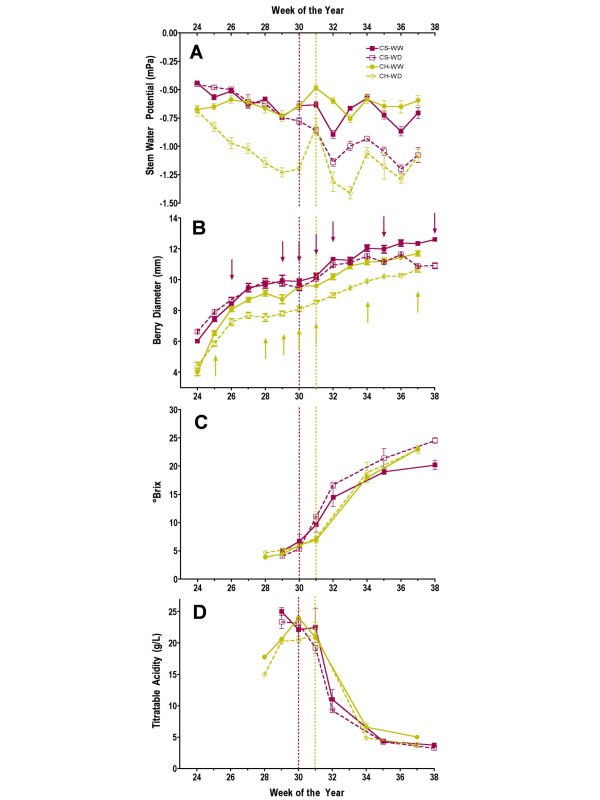
**The effects of water deficit on various grape vine and berry parameters over time**. A) stem water potential, B) berry diameter, C) berry soluble solids, D) berry titratable acidity. Vertical magenta and green dotted lines mark the boundaries for véraison for Cabernet Sauvignon and Chardonnay, respectively. Arrows mark sampling dates for molecular analyses. Symbols represent means ± SE; n = 6. CH = Chardonnay, CS = Cabernet Sauvignon, WW = well watered, WD = water deficit.

Different ripening parameters (berry size, soluble solids, titratable acidity) were measured throughout development in Cabernet Sauvignon and Chardonnay berries in both well-watered (WW) and water deficit (WD) treatments (Fig. 1). Water deficit was administered simply by withholding water. Thus, these vines developed water-deficit at the rate natural to their local conditions. Note that Chardonnay and Cabernet Sauvignon had different flowering and harvest dates due to differences in genotype and location.

Stem water potentials were monitored in both cultivars, which were at different vineyards, in order to define water status conditions throughout the season and its impact on these ripening parameters. Water was applied to both WW and WD vines by drip irrigation, once the desired stem water potential was reached (-0.6 or -1.2 MPa, respectively). For Chardonnay, irrigation was initiated on week 24 and 29 for WW and WD vines, respectively. For Cabernet Sauvignon, irrigation was initiated on week 27 and 36 for WW and WD vines, respectively. Once started, irrigation was applied weekly, because there was no rainfall throughout the growing season.

Differences in the stem water potentials between WW and WD vines were detected by week 25, early in Chardonnay berry development (Fig. 1A) just after the administration of the water deficit treatment. This difference was maintained throughout the season until harvest. In contrast, the differences in stem water potentials between WW and WD Cabernet Sauvignon vines were not observable until week 30 and reached a minimum of -1.25 MPa at week 36 in WD vines. The slower rate of stress development for Cabernet Sauvignon could be due to a number of factors including differences in soil type, vapor pressure deficits, temperature, age of the vines, root stock, depth of roots, and trellising systems. In Chardonnay, the berry diameter was smaller in berries from WD plants over the course of berry development; differences ranged from 0.5 cm to 1 cm at the harvest stage (Fig. 1B). In contrast, a decrease in berry diameter of Cabernet Sauvignon under water deficit conditions was detectable first at the start of véraison (week 30; Fig. 1B).

Soluble solids content (°Brix) and titratable acidity (TA) were significantly affected in WD vines (p < 0.001) with varying results (Fig. 1C). °Brix increased in both Chardonnay and Cabernet Sauvignon berries for all treatments starting at véraison. There was no significant effect of water deficit on the start of véraison. °Brix was increased significantly (p = 0.0002) in Cabernet Sauvignon berries of WD vines compared to berries of WW vines starting one week after véraison (Fig. 1C), however there was no significant effect (p = 0.26) of water deficit on the °Brix of Chardonnay berries. TA (Fig. 1D) was highest just prior to véraison, and then decreased until harvest in both cultivars. WD significantly reduced TA in Chardonnay berries (p < 0.01) but not in Cabernet Sauvignon berries (p = 0.15).

### Functional comparison of genes sets differentially expressed in both cultivars during water deficit

The effect of water deficit on the global gene expression of berries of both cultivars was examined. Total RNA was extracted from the berries at seven different time points for each cultivar and treatment as indicated in Figure 1B. Global gene expression analysis was performed using the Affymetrix *Vitis *Genome Array. Out of the 16,436 *Vitis *probe sets placed on the array, 10,101 probe sets were detected in at least two of three replicates within every experimental condition (water status, time point, and cultivar) according to the GCOS software (Affymetrix, USA) leading to an overall specialized detection rate of 61.7%. To validate expression profiles obtained by the *Vitis *Genome Array, quantitative real-time RT-PCR (qRT-PCR) was performed on four different transcripts (two for each cultivar) that increase and decrease in abundance using gene-specific primers (Additional file 1). Transcript abundance patterns were calculated for both cultivars and treatments along the entire course of berry development. Linear regression analysis resulted in an r^2 ^of 0.89 indicating a very good correlation between transcript abundance assessed by qRT-PCR and the expression profiles obtained with the *Vitis *Genome Arrays (Additional file 2). This is consistent with previous results from these arrays [21].

**Figure 2:**
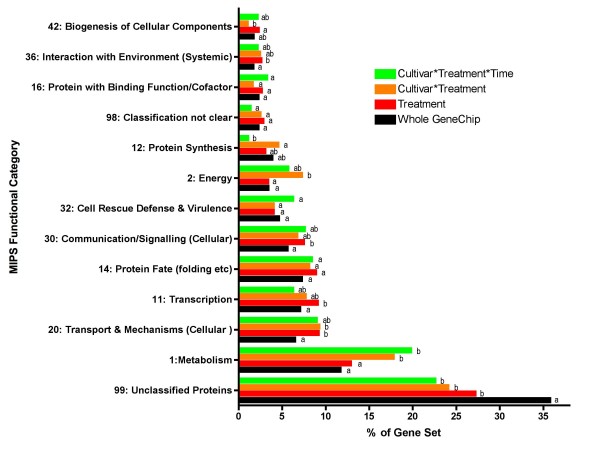
**The percentage of genes in a functional category for each significant effects group compared to the entire set on the *Vitis vinifera *(Grape) Genome Array**. Significant differences between groups were determined by a chi-square test and a Fischer's exact test. Columns with the same letters next to the column indicate that there were no significant differences between that group and the other group within each functional category. Different letters indicate significant differences within that functional category. When multiple letters are present (e.g. ab) then this column is not statistically different from columns with either of these letters (i.e. a or b).

A simple, three-way fixed effects ANOVA was performed, to examine probe sets with a significant treatment effect, a significant treatment and cultivar interaction effect, and a significant treatment, cultivar and time interaction effect. There were 3819, 2036, and 368 significant probe sets (p = 0.05) in each effect test, respectively (Additional files 3, 4, and 5). The genes represented by these probe sets were functionally categorized and compared to the functional categorization for the entire *Vitis *Genome Array (Fig. 2). Functional categories were assigned using the Munich Information Center for Protein Sequences (MIPS, ver. 2.1) catalog of top Arabidopsis BLAST hits [22]. Because we detected some errors in the functional annotation for some Unigenes, functional categorization of each Unigene was verified or corrected manually using BLAST functions at PLEXdb . A complete list of functional categories for all probe sets on the *Vitis *Genome Array has been constructed and is available at the grape annotation page of this website.

Water deficit had significant effects on several functional categories including transport, transcription, and signaling categories (Fig. 2). Chi-square and Fisher's exact tests showed a statistically significant increase in the percentage occurrence of genes in these categories. There was a significant treatment and cultivar interaction effect on the metabolism, transport and energy categories when compared to all probe sets on the *Vitis *Genome Array. Likewise, genes with a significant treatment, cultivar and time interaction effect had more significant increases in abundance of transcripts related to metabolism than the treatment-significant set as well as for all probe sets on the array. The metabolism category had the largest differences in transcripts that were significantly increased in the treatment and cultivar interaction effect and therefore will be the focus in the remainder of this paper.

### Interactive effects of water deficit and genotype on berry metabolism

**Figure 3:**
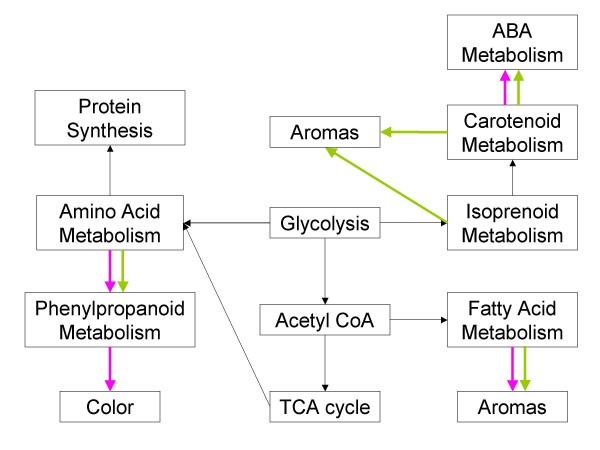
**The influence of water deficit on the metabolism in grapes**. The thicker colored arrows indicate that there were significant effects of water deficit on the particular processes linked by the arrows. Magenta arrows represent significant changes for Cabernet Sauvignon and green arrows represent significant changes for Chardonnay.

A number of maps of metabolic pathways were created by matching probe sets on the *Vitis *Genome Array to enzymes in known metabolic pathways (unpublished results). Figure 3 summarizes how water deficit affected the major metabolic pathways and their interrelationships for each cultivar. Bold colored arrows indicate those pathways and interrelationships most affected by water deficit in Cabernet Sauvignon (magenta) and Chardonnay (green), including ABA, carotenoid, amino acid, fatty acid and phenylpropanoid metabolism. Water deficit responses differed significantly between the two cultivars. Some components of these pathways were increased and others were decreased. For example, water deficit affected carotenoid, ABA, amino acid and fatty acid metabolism in both cultivars, but the specific effects in these pathways were different for each of the cultivars. In other cases, only one cultivar was significantly affected by water deficit (e.g. isoprenoid metabolism in Chardonnay). Some of the specific details of these differences are described in the following sections.

#### ABA metabolism

**Figure 4:**
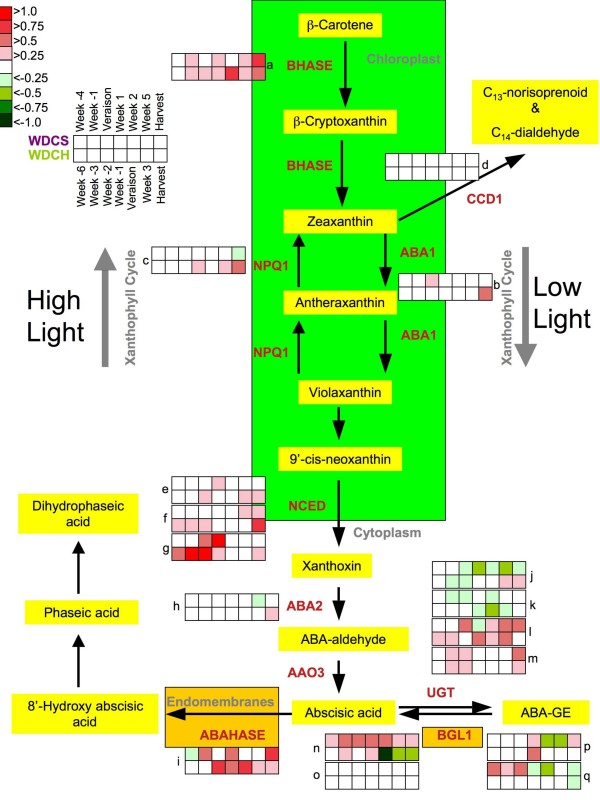
**The effect of water deficit on ABA metabolism**. The color in each box next to the enzyme name represents the ratio of the log2 of the transcript abundance of WD/WW. The upper set of boxes is for Cabernet Sauvignon and the lower set is for Chardonnay at various times pre- or post-véraison as indicated by the key. Heatmaps of the time series represent changes in gene expression levels as indicated by the color legend above. The high and low light arrows show the direction of the pathway under each condition, respectively. a) BHASE (β-carotene hydroxylase), 1617541_s_at; b) ABA1 (zeaxanthin epoxidase), 1606913_at; c) NPQ1 (violaxanthin de-epoxidase), 1615888_at; d) CCD1 (9,10 [9',10']carotenoid cleavage dioxygenase), 1610743_s_at; e) NCED (nine-cis-epoxycarotenoid dioxygenase), 1607029_at; f) NCED, 1610455_at; g) NCED, 1608022_at; h) ABA2 (xanthoxin dehydrogenase), 1606686_at; i) ABAHASE ((+)-abscisic acid 8'-hydroxylase), 1621989_at; j) UGT (UDP-glucose glucosyltransferase), 1621418_at; k) UGT, 1621051_at; l) UGT, 1615714_at; m) 1618155_at; n) BGL1 (β-glucosidase), 1622281_at; o) BGL1, 1608202_at; p) BGL1, 1620808_at; q) BGL1, 1620679_at.

There were significant effects of water deficit on the transcript profiles of genes involved in ABA metabolism (Fig. 4). The profiles of the log_2 _ratio (WD/WW) of the transcript abundance of the genes (probe sets) matched to each enzyme in the ABA metabolic pathway are shown as heat maps (Fig. 4). Boxes from left to right follow berry development over time. The upper set of boxes is for Cabernet Sauvignon and the lower set is for Chardonnay for each probe set. A number of genes were mapped to multiple probe sets on the *Vitis *Genome Array; in some cases, heat boxes for multiple probe sets were displayed for each enzyme in order to identify transcripts that might participate more in a particular pathway or process. There were significant effects of water deficit on ABA metabolism for both cultivars. Water deficit increased significantly (p < 0.0001) the transcript abundance for both cultivars of important regulatory enzymes in the ABA metabolic pathway: β-carotene hydroxylase (BHASE), nine-cis-epoxycarotenoid dioxygenase (NCED) and (+)-abscisic acid 8'-hydroxylase (ABAHASE). There were small but significant effects (p < 0.0001) of water deficit on xanthoxin dehydrogenase (ABA2). The water-deficit responsive transcript profiles displayed for UDP-glucose glucosyltransferase (UGT) and β-glucosidase (BGL1) should be viewed with caution, as these have not yet been clearly identified with ABA-specific functions in grape. However, these profiles are useful to identify candidate genes for further testing.

**Figure 5:**
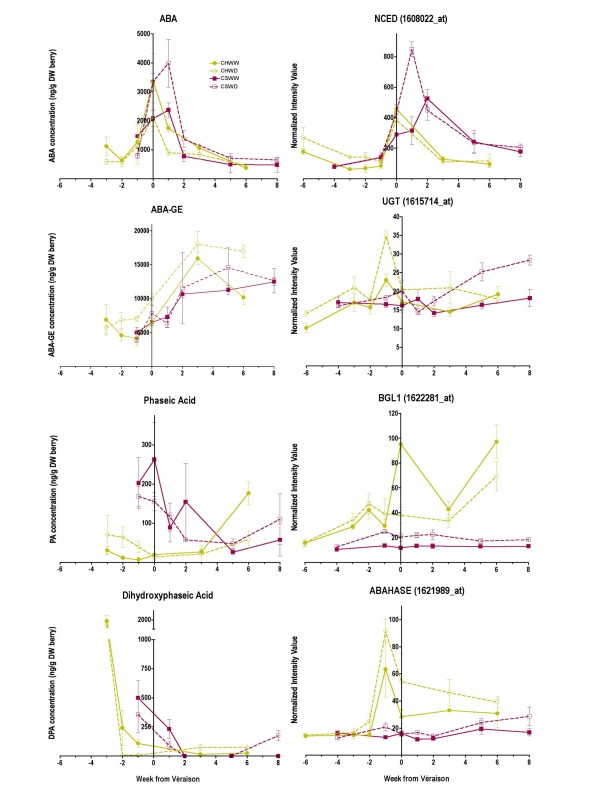
**The influence of water deficit on metabolites and related transcripts of enzymes affecting ABA metabolism**. The lines and symbols are the same as Figure 1; n = 3. ABA (abscisic acid); ABA-GE (abscisic acid glucose ester); NCED (nine-cis-epoxycarotenoid dioxygenase), 1608022_at; UGT (UDP-glucose glucosyltransferase), 1615714_at; BGL1 (β-glucosidase), 1622281_at; ABAHASE ((+)-abscisic acid 8'-hydroxylase), 1621989_at.

The concentrations of ABA and its metabolites varied across berry development and were influenced by water deficit (Fig. 5). Water deficit increased ABA concentrations significantly (p = 0.01) in Cabernet Sauvignon at véraison and one week following véraison. In contrast, there was a significant decrease in ABA concentrations (p = 0.044) at and one week after véraison in WD Chardonnay berries. ABA concentrations peaked at or one week after véraison in Chardonnay and Cabernet Sauvignon berries, respectively (Fig. 5). ABA concentrations were highly correlated (r = 0.65, p = 0.0007) with the transcript abundance (Fig. 5) of one particular NCED Unigene (*NCED1*, 1608022_at, TC57089) indicating that ABA concentrations in berries in general might be regulated primarily by this gene in berries and not by ABA from some external source such as leaves or roots (Fig. 5). However, additional ABA metabolism steps might contribute significantly to the reduced ABA concentrations in Chardonnay berries (see Fig. 5 and discussion below).

ABA can be metabolized into ABA-glucose ester (ABA-GE) and phaseic acid, which is then reduced to dihydrophaseic acid. ABA-GE concentrations were relatively constant before véraison, but there was a trend towards increasing ABA-GE concentrations after véraison. Water deficit significantly increased ABA-GE concentrations (Fig. 5) in Chardonnay berries (p = 0.0005) but not in Cabernet Sauvignon (p = 0.59). There are a number of transcripts of UDP-glucose glucosyltransferases represented on the *Vitis *Genome Array. None have been clearly defined as encoding the enzyme involved in ABA glycosylation. However, the steady-state abundance of one transcript (1615714_at, CF404835) was increased by water deficit in both cultivars (Fig. 5). This transcript was significantly correlated with the concentrations of ABA-GE in Cabernet Sauvignon (p = 0.0292), but not for Chardonnay (p = 0.247). Thus, this hypothesis needs additional testing before sound conclusions can be drawn.

β-glucosidases may be involved in the cleavage of glucose from ABA-GE converting it to ABA. None of the profiles of candidate β-glucosidases on the *Vitis *Genome Array (Fig. 4 and 5) significantly correlated with either ABA or ABA-GE concentrations indicating that either there is another candidate that is not on the *Vitis *Genome Array or that this enzyme's activity is regulated by post-translational modifications.

ABAHASE is the enzyme that catabolizes ABA into 8'-hydroxy ABA, which is nonenzymatically converted to phaseic acid, which then can be reduced to dihydrophaseic acid. Water deficit slightly increased the concentration of dihydrophaseic acid in both Cabernet Sauvignon and Chardonnay berries at maturity, but these effects were not statistically significant (Fig. 5). Water deficit had no significant effect on the concentrations of phaseic acid for either cultivar (Fig. 5). The transcript abundance of *ABAHASE *was greater in Chardonnay than in Cabernet Sauvignon and was increased significantly by water deficit for both cultivars (p = 0.0176 and 0.0108, respectively). There was no correlation of the transcript abundance of *ABAHASE *with concentrations of phaseic acid. There was a substantial increase in transcript abundance of *ABAHASE *at véraison for Chardonnay, but not for Cabernet Sauvignon. The lack of correlation could be due to post-translational modifications or to its regulation being associated with some other transcript.

**Figure 6:**
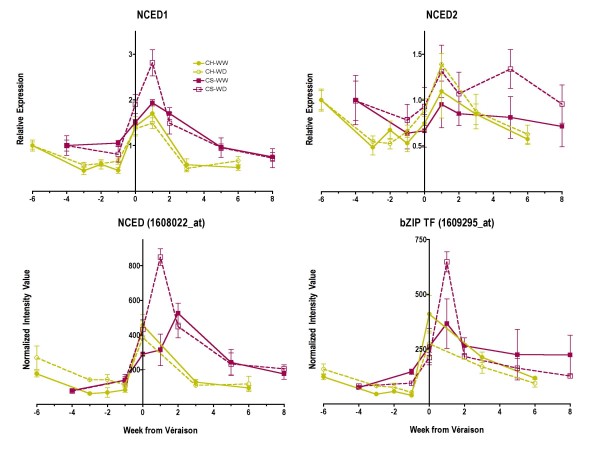
**The influence of water deficit on transcript abundance *NCED1 *and *NCED2 *using qRT-PCR and other ABA-related transcripts on the *Vitis *Genome Array**. Data were normalized to the transcript abundance of an ankyrin-repeat protein (1612584_s_at) that did not change with developmental stage or stress treatment. The lines and symbols are the same as Figure 1; n = 3. NCED1 (nine-cis-epoxycarotenoid dioxygenase 1), 1608022_at, TC57089, AY337613; NCED2 (nine-cis-epoxycarotenoid dioxygenase 2), TC71235, AY337614; bZIP TF (homeobox-leucine zipper transcription factor), 1609295_at.

In a recent study [23], transcript abundance of *NCED2*, but not *NCED1*, was shown to increase at ripening initiation or véraison in Cabernet Sauvignon berries. This prompted us to reexamine the probe set (1608022_at) on our microarray. When the probe set sequence was compared with the grape genome in the nr database at NCBI, there was 100% identity with *NCED1 *and 76% identity with *NCED2*, which had no probe set match on the array. To be certain that the data for 1608022_at was that of *NCED1*, qRT-PCR was used on another set of berry extractions with the same primers as in the previous study [23]. The transcript abundances of *NCED1 *and not *NCED2 *using qRT-PCR (Fig. 6) were correlated clearly with the probe set data and ABA concentrations.

Interestingly, the mRNA expression of a bZIP transcription factor (1609295_at, TC59629) that is similar to *ATHB-7 *in Arabidopsis [24], was highly correlated (r = 0.86, p = 0.0001) with *NCED1 *expression on the *Vitis *Genome Array (Fig. 6). The transcript abundance of this transcription factor in Arabidopsis is increased by water deficit [24], inhibits growth, operating downstream in response to elevated ABA concentrations [25].

#### Carotenoid metabolism and light stress

**Figure 7:**
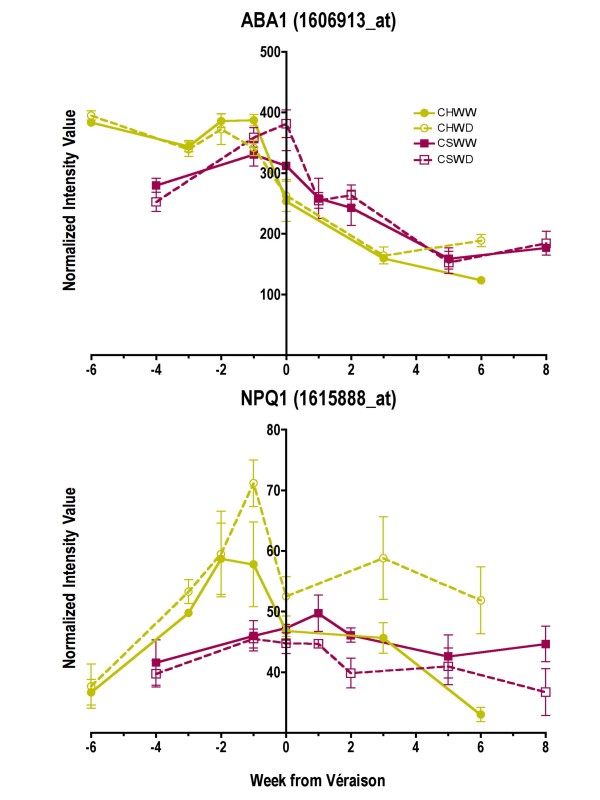
**The influence of water deficit on the transcript abundance of two enzymes in the carotenoid pathway**. The symbols are the same as Figure 1; n = 3. ABA1 (zeaxanthin epoxidase), 1606913_at; NPQ1 (violaxanthin de-epoxidase), 1615888_at.

**Figure 8:**
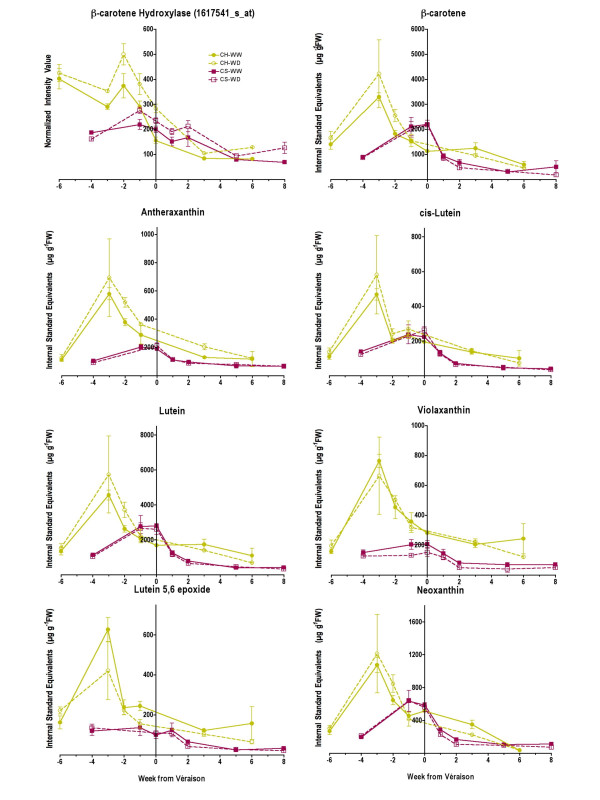
**Comparison of the effects of water deficit on the abundance of β-hydroxylase transcript with detectable carotenoid metabolites**. The symbols are the same as in Figure 1; n = 6 for carotenoids; n = 3 for transcripts.

ABA is synthesized from carotenoids and some transcripts in the carotenoid pathway are also affected by water deficit (Fig. 4). BHASE acts in two parallel pathways that lead to the conversion of β-carotene into zeaxanthin eventually leading to ABA, and α-carotene into zeinoxanthin eventually leading to lutein. These carotenoids can act as photoprotectants under high light intensities [26,27]. The transcript abundance of *BHASE *was increased by water-deficit in both cultivars (Fig. 4 and 8). The transcript abundance of zeaxanthin epoxidase (*ABA1*; Fig. 7) was not significantly increased in WD berries in either Chardonnay or Cabernet Sauvignon (p = 0.66 and 0.25, respectively). However, in later weeks, the transcript abundance of violaxanthin de-epoxidase (non-photochemical quenching 1, *NPQ1*) was significantly increased (p = 0.0018) in WD Chardonnay and decreased (p = 0.0014) in WD Cabernet Sauvignon relative to their respective controls (Fig. 7). Conversion of violaxanthin to zeaxanthin by violaxanthin de-epoxidase is required for the dissipation of excess light energy in the antennae of photosystem II [28]. These results indicate that WD Chardonnay berries may be undergoing significantly more light stress than WD Cabernet Sauvignon berries, presumably due to a lack of anthocyanin-derived photoprotective pigments.

To confirm these results, carotenoid concentrations were measured by HPLC in the berries of both cultivars at all 7 time points. The two most abundant carotenoids were lutein and β-carotene (Fig. 8, Additional file 6). There were also detectable levels of neoxanthin, violaxanthin, lutein 5,6 epoxide, antheraxanthin, and cis-lutein in both cultivars (Additional file 6). Zeaxanthin was detectable but not resolvable on the chromatograms for either cultivar. The relatively low levels of chlorophyll *a *and corresponding increase in pheophytins (Additional file 6) were artifacts of the extraction protocol that did not alter carotenoid levels, but did alter chlorophylls. Carotenoid concentrations were generally 2-fold or higher in Chardonnay than in Cabernet Sauvignon for most carotenoids and developmental stages (Fig. 8). This result was consistent with the higher transcript abundance of *BHASE *(Fig. 8) contributing to higher carotenoid biosynthesis and the lower transcript abundance of *NCED *contributing to carotenoid catabolism (Fig. 6) for Chardonnay relative to that of Cabernet Sauvignon. Most carotenoid concentrations were not affected significantly by water deficit for either cultivar. However, water deficit increased significantly (p = 0.0041) the antheraxanthin concentration before and after véraison in Chardonnay berries (Fig. 8). The correlation of the *BHASE *transcript abundance with the antheraxanthin concentration was not significant at the 95% confidence level (r = 0.51, p = 0.06). However, if the first time point is removed from the analysis, then the correlation becomes highly significant (r = 0.87, p = 0.0002). In Cabernet Sauvignon, the violaxanthin concentration was decreased significantly (p = 0.0007) by water deficit, consistent with the increased *NCED *transcript abundance that would accelerate its catabolism.

#### Photosynthesis and glycolysis

**Figure 9:**
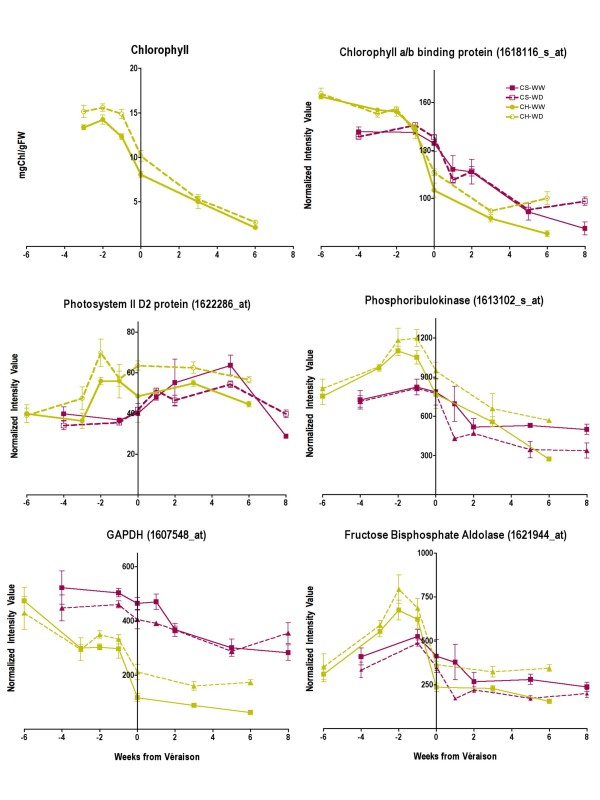
**The influence of water deficit on chlorophyll and genes involved in photosynthesis and glycolysis**. The symbols are the same as in Figure 1; n = 6 for chlorophyll; n = 3 for transcripts. GAPDH (NADP-dependent glyceraldehyde-3-phosphate dehydrogenase), 1607548_at.

There was evidence that photoinhibition was induced by water deficit in Chardonnay berries. This hypothesis was supported by the response of genes involved in the photosystem II electron transport chain, RUBP regeneration and glycolysis (Fig. 9). Chlorophyll concentrations of Chardonnay berries were significantly increased (p < 0.001) by water deficit before véraison, but there were no significant differences after véraison. The transcript abundance of chlorophyll a/b binding protein for Chardonnay follows the same pattern as chlorophyll over time. In contrast, the transcript abundance of several genes in Chardonnay berries was increased significantly (p < 0.001) by water deficit after véraison. These include photosystem II D2 protein, phosphoribulose kinase, glyceraldehydes-3-phosphate dehydrogenase (*GAPDH*) and fructose bisphosphate aldolase (*FBA*) (Fig. 9). The increased transcript abundances of these photosynthetic and glycolytic genes are likely indicators of repair responses to photoinhibition during water deficit [29]. The transcript abundance of the same genes in Cabernet Sauvignon berries are generally decreased or unaffected by water deficit (Fig. 9).

#### Isoprenoid, carotenoid, and fatty acid metabolism involved in volatile production

**Figure 10:**
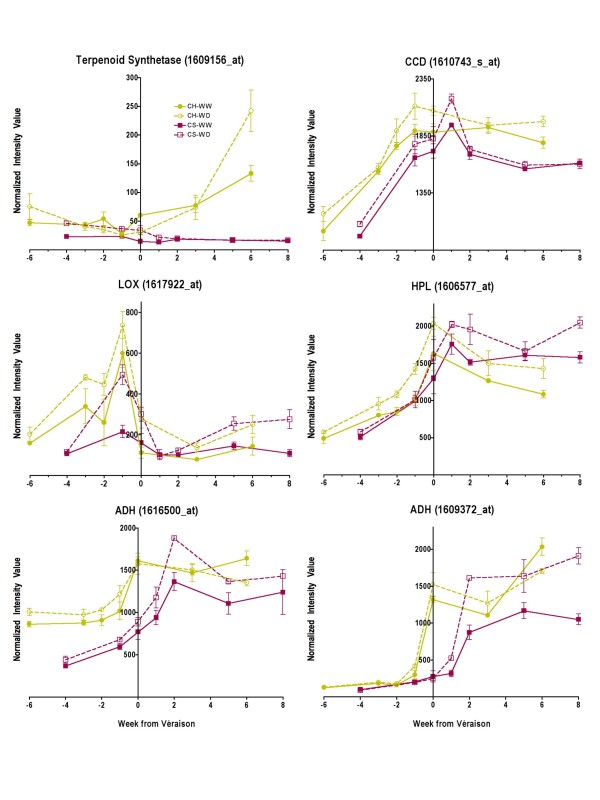
**The influence of water deficit on the transcript abundance of enzymes involved in carotenoid and fatty acid metabolism**. The symbols are the same as in Figure 1; n = 3. CCD (9,10 [9',10']carotenoid cleavage dioxygenase), 1610743_s_at; LOX (lipoxygenase), 1617922_at; HPL (hydroperoxide lyase), 1606577_at; ADH (alcohol dehydrogenase), 1616500_at and 1609372_at).

Water deficit increased the transcript abundance of enzymes involved in volatile compound production (Fig. 10). The transcript abundance of one terpenoid synthetase was increased significantly in WD Chardonnay (p = 0.047) at maturity, but not Cabernet Sauvignon at the mature stage when the grapes were harvested. However, the transcript abundance was increased significantly in WD Cabernet Sauvignon at earlier stages (p < 0.0001). This transcript has been shown to be important in the production of sesquiterpenoids during the late ripening stages of Gewürztraminer, an aromatic white grape [30].

Downstream of BHASE, a carotenoid cleavage dioxygenase (CCD) known to increase at véraison [31], cleaves zeaxanthin into a C_13_-norisoprenoid and a C_14_-dialdehyde, both volatile compounds. Water deficit increased the transcript abundance of *CCD *in both cultivars. There was a significant increase at earlier berry stages in Cabernet Sauvignon (p = 0.0024), but there was no significant increase in transcript abundance above that of well-watered grapes at maturity (Fig. 10). However, there was a significant increase (p = 0.0007) in transcript abundance in WD Chardonnay berries at maturity. Zeaxanthin was unresolvable due to low concentrations. We do not know if zeaxanthin concentration is the limiting factor for this reaction. More research on the rate of supply of zeaxanthin, enzyme concentration and activity, and zeaxanthin concentrations at the site of action are needed to determine the limitations of CCD activity.

The transcript abundance of several different lipoxygenases (LOX) was increased significantly by water deficit in both cultivars. For example, the transcript abundance of one LOX (1617922_at) increased significantly (p < 0.0001, Fig. 10) at all stages of development in Chardonnay and at several time points in Cabernet Sauvignon, most importantly at maturity. These lipoxygenases convert the fatty acid, linolenic acid, to hydroperoxides [32,33], which through several other enzymatic steps can lead to the formation of volatile esters in wines. In the next step of this pathway, the hydroperoxides are converted to grassy-flavored volatile aldehydes like hexenal and hexenal-3-al by hydroperoxide lyase (HPL) [32]. The transcript abundance of this gene was also increased by water deficit for both cultivars (p < 0.0001) throughout most of berry development including at maturity (Fig. 10). Hexenal can be converted to hexanol, another grassy aroma, by alcohol dehydrogenases [34,35]. The transcript abundance for some of these enzymes was also increased in Cabernet Sauvignon berries by water deficit (Fig. 10). Alcohol acyl transferases (AAT) can convert alcohols to volatile esters (no clearly identified probe set exists on the *Vitis *Genome Array for *AAT *to test this hypothesis in this experiment). We have detected three-fold increases in a fruity aroma (hexyl acetate) in WD Chardonnay wines, which may be derived from this pathway [36]. Yeast metabolism during fermentation is likely to be a contributing factor in the production of this compound.

#### Proline and glutamate metabolism

**Figure 11:**
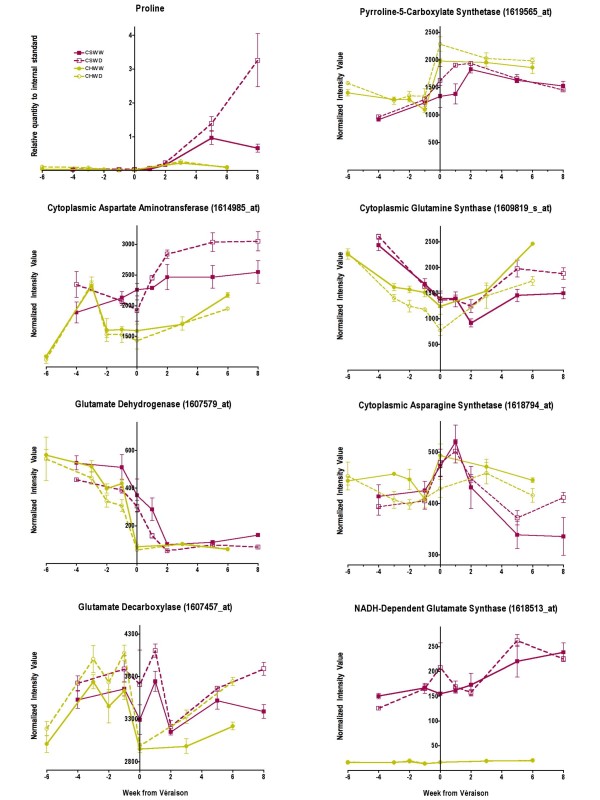
**The influence of water deficit on proline concentrations and the transcript abundance of enzymes involved in proline and glutamate metabolism**. The symbols are the same as in Figure 1; n = 6 for proline; n = 3 for transcripts.

The transcript abundance of genes involved in most amino acid metabolism pathways was differentially affected by water deficit between the cultivars (Additional file 4), including proline, glutamate, phenylalanine, tyrosine, methionine, cysteine, and glycine to name but a few. Proline is the most abundant amino acid in berries and can contribute to a sweet taste. Proline has many biological functions that include acting as an energy source, antioxidant and osmoprotectant [37,38]. Proline concentrations increased in berries at véraison in both cultivars (Fig. 11). There were substantially higher proline concentrations in Cabernet Sauvignon compared to Chardonnay at harvest. Water deficit significantly increased proline concentrations in Cabernet Sauvignon berries (p = 0.0004), increasing by 3-fold at maturity (Fig. 11). There was no significant effect (p = 0.2314) of water deficit on proline concentration in Chardonnay berries.

Δ^1^-pyrroline-5-carboxylate synthetase (P5CS) is considered the rate-limiting step in proline biosynthesis [37,38]. P5CS converts glutamate into glutamate 5-semialdehyde, which spontaneously converts to Δ^1^-pyrroline-5-carboxylate. Δ^1^pyrroline-5-carboxylate reductase (P5CR) catalyzes the last step in proline biosynthesis, converting Δ^1^-pyrroline-5-carboxylate into proline. There are no probe sets for *P5CR *on the *Vitis *Genome Array, but there are probe sets for *P5CS*. Water deficit increased the transcript abundance of *P5CS *in both Chardonnay (p = 0.0028) and Cabernet Sauvignon (p = 0.0258) berries at véraison, but no significant effects of water deficit were observed at maturity when proline concentration was most affected by water deficit in Cabernet Sauvignon (Fig. 11).

Glutamate is central to amino acid metabolism and nitrogen assimilation [37,38]. Key enzymes involved in glutamate metabolism are glutamate dehydrogenase (GDH), glutamate decarboxylase (GAD), glutamate synthase (GOGAT), glutamine synthetase (GS), aspartate aminotransferase (AspAT), and asparagine synthetase (AS). The enzymes are responsible for assimilating ammonium into glutamate, except for GAD, which irreversibly catabolizes glutamate into γ-aminobutyric acid (GABA).

Glutamate concentrations in the berry were too low to provide reliable quantitative data in the GC-MS chromatograms. However, the *Vitis *Genome Array data supported the hypothesis that glutamate metabolism contributes to the increased proline concentrations in WD Cabernet Sauvignon berries (Fig. 11). Water deficit increased significantly the transcript abundance of the cytoplasmic forms of *ASPAT *(p < 0.0001), *GS *(p = 0.0048), and *AS *(p < 0.0001) in Cabernet Sauvignon at maturity (Fig. 11). In contrast, water deficit had no effect on the transcript abundance of *ASPAT *(p = 0.1353) or it decreased the transcript abundance of *GS *(p < 0.0001) and *AS *(p = 0.0029) in Chardonnay berries at maturity. Water deficit had no statistically significant effect on the transcript abundance of *GDH *in Chardonnay (p = 0.0569), but significantly decreased the transcript abundance of *GDH *in Cabernet Sauvignon (p = 0.0014). There were no significant effects of water deficit on the transcript abundance of NADH-dependent GOGAT for either cultivar, but transcript abundance was much higher for Cabernet Sauvignon than Chardonnay (Fig. 11). Water deficit might have significantly increased glutamate catabolism, because the transcript abundance of *GAD *was increased significantly in Cabernet Sauvignon (p = 0.0014) and Chardonnay (p < 0.0001). The data indicate that *ASPAT *might have provided the greatest contribution to the increased proline concentrations in WD Cabernet Sauvignon berries, because there were large differences between Cabernet Sauvignon and Chardonnay (Fig. 11) relative to the other transcripts.

#### Phenylpropanoid metabolism

Polyphenolic compounds play an important role in the quality of grapes and wines, and the way these substances are transformed during vinification influences the quality of wine, directly or indirectly, conferring on it some of its structure and sensorial properties [39]. Phenolics are classified as nonflavonoid and flavonoid compounds and are derived from the phenylpropanoids. Many of these phenolics, including anthocyanins and flavonols, contribute to human-health benefits [40-42].

**Figure 12:**
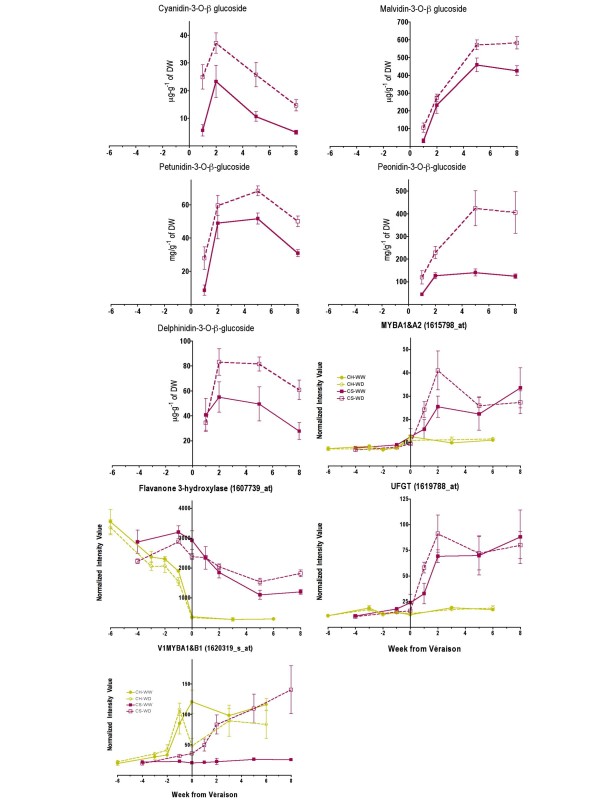
**The influence of water deficit on berry anthocyanin concentrations and transcripts associated with regulation and metabolism in the anthocyanin pathway**. The symbols are the same as in Figure 1; n = 6 for anthocyanins; n = 3 for transcripts. UFGT (UDP-glucose 3-O-flavonoid:glucosyltransferase) 1619788_at; MYBA1&A2 (*Vitis vinifera *MYB transcription factor A1 and *Vitis vinifera *MYB transcription factor A2), 1615798_at cross hybridizes to the transcripts of both transcription factors; VlMYBA1&B1 (*Vitis labrusca *MYB transcription factor A1 and *Vitis labrusca *MYB transcription factor B1) 1620319_s_at cross hybridizes to the transcripts of both transcription factors.

Recently, water deficit was shown to accelerate ripening and induce changes in gene expression regulating flavonoid biosynthesis, especially the anthocyanin pathway as measured by the increase in total anthocyanins [9]. Our results on individual anthocyanins were consistent with this study; water deficit increased approximately 2-fold the accumulation of the five major anthocyanins in Cabernet Sauvignon berries relative to berries from well-watered vines (Fig. 12). The concentration of all anthocyanins increased rapidly after véraison.

Anthocyanin biosynthesis is under the control of two MYB transcription factors, MYBA1 and MYBA2 [43-45]. Both transcription factors regulate UDP-glucose 3-O-flavonoid:glucosyltransferase (UFGT) in a similar manner [45]. UFGT catalyzes one of the last steps in anthocyanin biosynthesis. On the *Vitis *Genome Array, there is a probe set (1615798_at) that hybridizes to the transcripts of both transcription factors. The transcript abundance for these transcription factors was increased by water deficit and also was correlated highly with *UFGT *transcript abundance (Fig. 12). The rapid increase in transcript abundance of these genes after véraison correlated well with the rapid increase in the individual anthocyanins during the ripening phase (Fig. 12). Malvidin 3-O-β-glucoside was the most abundant anthocyanin under well-watered conditions, being 4-fold higher than the next most abundant anthocyanin, peonidin 3-O-β-glucoside. While all five anthocyanins were increased by water deficit, peonidin 3-O-β-glucoside was increased the most (4-fold as compared to the 50% increase for malvidin 3-O-β-glucoside). Peonidin gives purplish-red hues to grapes, whereas malvidin gives red hues. The increase in anthocyanins and their overall change in composition in the berries undoubtedly contributed to the changes in color that are observed in Cabernet Sauvignon wines made from WD berries [8,36], including changes in hue, chroma and luminosity [36].

Another transcript that functions in an earlier step in the flavonoid pathway, flavanone 3-hydroxylase, was also affected by water deficit, but at a later stage of development (Fig. 12). The transcript abundance of other Unigenes related to MYB transcription factors and potentially related to the regulation of the anthocyanin pathway in plants was increased in WD Cabernet Sauvignon berries compared to WW berries (Additional file 3). One example (1620319_s_at, TC61048) encodes *VlMYBB1*, whose function has been reported previously in grape berry [44]. Transient expression of the *VlMYBB1 *cDNA in grape cells did not result in red pigment accumulation in the vacuole suggesting that this transcription factor was not directly involved in the regulation of the *UFGT *gene. UFGT catalyzes one of the last steps of color (anthocyanin) development in grape berry skin [44]. The transcript abundance of this Unigene was increased in WD Cabernet Sauvignon berries (Fig. 12).

**Figure 13:**
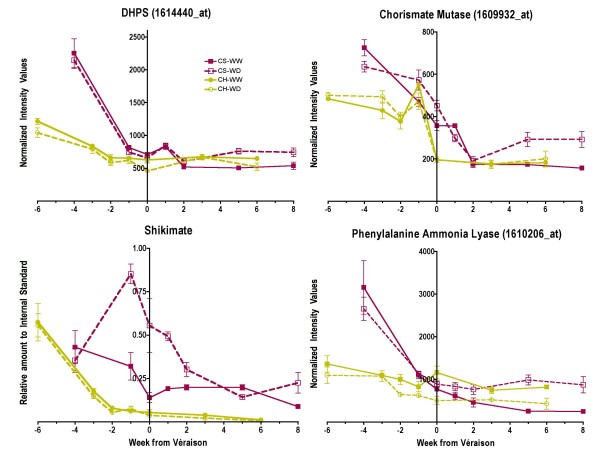
**The influence of water deficit on the transcript abundance of enzymes involved in the shikimate and early stages of the phenylpropanoid pathways**. The symbols are the same as in Figure 1; n = 6 for shikimate; n = 3 for transcripts. DHPS (3-deoxy-D-arabino-heptulosonate 7-phosphate synthase), 1614440_at.

Water deficit also increased shikimate concentrations and the steady-state mRNA abundance of genes encoding key enzymes at earlier steps in the shikimate and phenylpropanoid pathway in Cabernet Sauvignon (Fig. 13). For instance, the transcript abundance of 3-deoxy-D-arabino-heptulosonate 7-phosphate synthase (*DHPS*, 1614440_at, TC54321), chorismate mutase (1609932_at, TC53641) and phenylalanine ammonia lyase (1610206_at, TC69585) was significantly increased (p < 0.01) in WD Cabernet Sauvignon. In contrast, the transcript abundance of these genes was not increased by water deficit in Chardonnay. In addition, shikimate concentrations were increased by WD in Cabernet Sauvignon, but not in Chardonnay.

**Figure 14:**
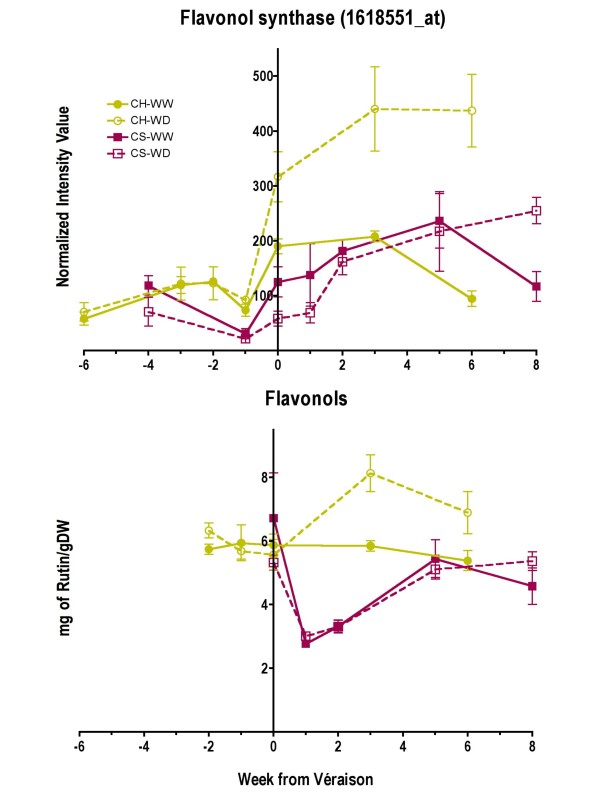
**The influence of water deficit on berry total flavonol concentrations and the transcript abundance of flavonol synthase**. The symbols are the same as in Figure 1; n = 6 for flavonols; n = 3 for the flavonol synthase transcript, 1618551_at.

Flavonols contribute to the bitter taste and color of red wine by stabilizing anthocyanin pigments [39]. In grape berries, they also play a role as UV protectants [46]. Increasing light intensity enhances flavonol accumulation in grapes [47,48]. Flavonol synthase (FLS), which catalyzes the reaction from dihydroflavonol to flavonol, is regarded as one of the main enzymes involved in flavonol biosynthesis [49,48]. The transcript abundance of a flavonol synthase (1618551_at, TC60553) named *FLS4 *[48] increased (p < 0.0001) across berry development in WD plants compared to WW plants in Chardonnay (Fig. 14) and coincided with differences (p = 0.0052) in the amount of total flavonols observed in Chardonnay berries at harvest stage (Fig. 14). These increases were larger for Chardonnay compared to Cabernet Sauvignon during the last two stages of berry development.

#### Sugar, ABA and anthocyanin metabolism

**Figure 15:**
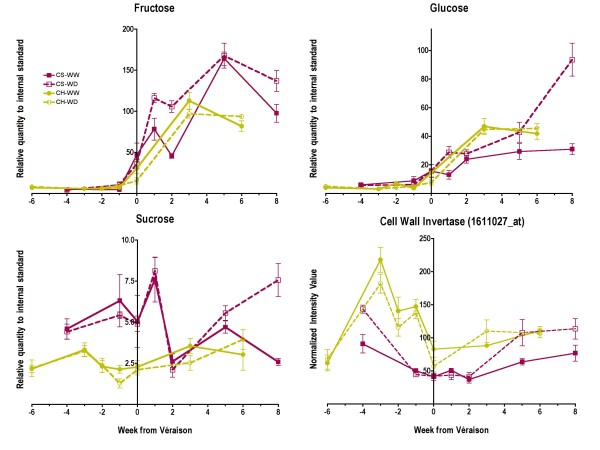
**The influence of water deficit on sugar accumulation and the transcript abundance of a cell wall invertase**. The symbols are the same as in Figure 1; n = 6 for sugars; n = 3 for the cell wall invertase transcript, 16111027_at.

As mentioned previously, ABA and anthocyanin metabolism are closely associated with sugar metabolism and transport. What is the timing of these three events? Do they occur simultaneously or does one event precede another? Accumulation of both fructose and glucose is first perceptible at véraison in Cabernet Sauvignon and Chardonnay (Fig. 15). ABA concentrations in both cultivars start to increase just prior to véraison (Fig. 6). Changes in sucrose accumulation or the transcript abundance of a cell wall invertase (1611027_at) were not well correlated with fructose and glucose accumulation (Fig. 15) indicating that other invertases were likely involved or that the enzyme was regulated by post-transcriptional modifications. In Cabernet Sauvignon, anthocyanins clearly accumulated at the start of véraison along with the transcript abundance of *MYBA1*, *MYBA2 *and *UFGT *(Fig. 12). Thus, it would appear that ABA concentrations increased in berries just prior to that of hexose accumulation in both cultivars and anthocyanins in Cabernet Sauvignon.

## Discussion

Cabernet Sauvignon (red-wine grape) and Chardonnay (white-wine grape) are two of the most widely grown cultivars of *Vitis vinifera *throughout the world and have different genetic backgrounds. Their genetic pedigree has been clearly established. Cabernet Sauvignon is the progeny of two French Bordeaux cultivars, Cabernet Franc (red) and Sauvignon Blanc (white) [50] and Chardonnay is the progeny of Pinot (red) and Gouais Blanc (white) that were widely grown in Northeastern France [51].

Most white grapes, including Chardonnay, evolved apparently from the mutation of two MYB transcription factors, *VvMYBA1 *and *VvMYBA2*, which regulate anthocyanin biosynthesis in the grape skin; Cabernet Sauvignon is heterozygous for red berry color having both a red and a white allele [45]. In the same paper it was shown that in Shiraz (red grape), the transcript abundance of these transcription factors along with UFGT, an enzyme involved in one of the last steps in anthocyanin biosynthesis, was increased at véraison, the beginning of fruit ripening and coloring [45].

In a previous greenhouse study, Cabernet Sauvignon shoot growth was more sensitive to water deficit and salinity than the shoot growth of Chardonnay [52]. In stressed plants, there was a reduction of the amount of proteins involved with photosynthesis, protein synthesis, and protein destination and this was correlated with the inhibition of shoot elongation. Many of the proteins that were increased by osmotic stress in Chardonnay were of unclassified or of unknown function, whereas proteins specifically increased by dehydration in Cabernet Sauvignon were involved in protein metabolism.

In this study, the response of these two cultivars to water deficit was evaluated. Given that they have different genetic backgrounds, different color compositions in their berry skins, and known differences in their tolerance to water deficits, we expected that there would be distinct differences in their metabolic responses to water deficit. Analysis of this water deficit study was confounded by the fact that the cultivars were studied at different vineyard sites. Any field study is imperfect in that one cannot control all experimental variables in the field. For example, the soil in a field is not uniform; there is natural soil variability within the site; therefore, the roots of each plant in a field might be exposed to a different soil environment. Pot experiments in the greenhouse also suffer from their own set of experimental limitations (e.g. root restriction, filtered light, unrealistic and rapid soil drying, etc.). In this study, not only were the cultivars exposed to slightly different climatic conditions, but also they were grown in different soils with different rooting depths. Consequently, the rate of application of water deficit for Cabernet Sauvignon was slower than the rate of application of water deficit for Chardonnay. The rate of decreasing water deficit could not be controlled, but the final level of water deficit reached was controlled. The timing and rate of water deficit could affect the stress response of the vines as discussed in the introduction. A gradual application of water deficit or salinity to grapevines allowed more time for more complex responses and possible acclimation than osmotic shock treatments [53]. Thus, while both cultivars were exposed to water deficit, there may be confounding factors in the field that limit our ability to make firm conclusions.

Nevertheless, some interesting observations have been made. For example, given that there was a faster rate of development of water deficit in Chardonnay and that the overall decrease in stem water potential was at least that of Cabernet Sauvignon relative to its control over the entire course of the season (Fig. 1), it would be expected that Chardonnay would have some water deficit responses that were more rapid and at least equal to that of Cabernet Sauvignon. For example, the induction of elevated ABA concentrations in Chardonnay would be expected to be at least as high as Cabernet Sauvignon, because elevated ABA concentrations are generally proportional to the water deficit level imposed on the plant [54-56] and ABA concentrations can be increased with osmotic shock or with slower applications of stress [57]. However, while ABA concentrations increased early on in Chardonnay, the ABA response to water deficit at véraison or one week after véraison was much more significant for Cabernet Sauvignon than Chardonnay indicating that these diverse responses might be due to varietal differences (Fig. 5). In the following sections, the impacts of water deficit on the most significant changes in metabolism that differ between these cultivars and how they impact important sensory attributes in grapes and wines are discussed.

### Is increased anthocyanin metabolism, proline and hexose accumulation regulated by ABA?

Our results (Fig. 12) are consistent with earlier reports on anthocyanin biosynthesis [45]; probe sets for both *VvMyBA1&2 *and *UFGT *increased significantly at véraison in Cabernet Sauvignon but not in Chardonnay. In addition, water deficit significantly increased mRNA expression of these genes at the start of véraison in Cabernet Sauvignon and was probably responsible for the most part in the increased concentrations of individual anthocyanins in the berries (Fig. 12) and in wine color [36]. Similar findings and conclusions were presented recently for berries of Cabernet Sauvignon [9], Merlot [15] and Agiorgitiko [58].

Coincident with these changes in anthocyanin accumulation, water deficit increased hexose (Fig. 15) and proline (Fig. 11) accumulation at véraison in Cabernet Sauvignon, but not in Chardonnay. Hexose accumulation is not only a function of biosynthesis (invertase activity), but also requires transport into the cells of the berries from the apoplast and may be dependent upon starch metabolism [21]. There are a number of hexose transporters that have been identified in grape berries [59]. A hexose transporter as well as an abscisic acid, stress, and ripening (ASR) protein are synergistically upregulated by ABA and sugar treatments [60,61]. Thus, not only might ABA trigger anthocyanin biosynthesis [16], but also hexose uptake. In addition, ABA is known to increase proline concentrations [62]. Our data were consistent with these hypotheses, but the temporal spacing of time points collected in this study was inadequate to properly resolve the timing of these events. However, support for this hypothesis can be found in another study on grape berries [63]. ABA was shown to stimulate invertase activity in the apoplast of grape berries and there was a rise of ABA concentration in the berry (with high time resolution) that clearly preceded the accumulation of soluble solids (hexoses) in the berries.

### ABA regulation during berry development

What is regulating the increase in ABA concentration prior to véraison in well-watered berries? NCED represents the first committed step in ABA biosynthesis [64] and its transcript abundance is often correlated with ABA concentrations. In addition, metabolic steps upstream of NCED and ABA catabolism downstream can also affect ABA concentrations in some tissues and organs [57].

Other reports are not consistent with our results or with each other. In a study on ripening Cabernet Sauvignon berries [23], *NCED2 *but not *NCED1 *was found to be up-regulated at véraison. However, ABA concentrations were not measured in that study, so the significance of this change in gene expression was not clear. We did observe a slight increase in transcript abundance of *NCED2 *(Fig. 7) at véraison, but ABA concentrations were correlated largely with the expression of *NCED1 *(Fig. 6).

In another study on Merlot berries [15], the transcript abundance of *NCED1 *and *NCED2 *was transiently increased by water deficit at véraison, but ABA concentrations also were not measured. These authors implied that NCED enzyme activity might not be significant based upon correlations of NCED transcript abundance with other ABA-regulated transcripts. In contrast to a previous study [23], the transcript abundance of *NCED2 *of the well-watered controls of Merlot berries did not increase before véraison but did so for *NCED1 *[15]. In our study we found that ABA concentrations of Cabernet Sauvignon and Chardonnay berries were highly correlated with *NCED1 *transcript abundance. While other mechanisms of ABA inactivation such as catabolism or glycosylation might affect ABA concentration, these mechanisms did not appear to be as significant as biosynthesis in our study.

What signals might regulate *NCED1 *transcript abundance? The transcript abundance of *NCEDs *and ABA concentration can be increased by water deficit [65]. Berry turgor declines at or before véraison as a result of solute accumulation in the apoplast [66,67] and could possibly be a trigger for the increase in ABA biosynthesis. However, turgor decreases appear to be the result of ABA-stimulated invertase activity and the subsequent significant accumulation of hexoses in the apoplast [68,67].

In addition, ABA concentrations are affected by ethylene [69] and phytochrome [70,71]. A recent study indicates that phytochrome can affect *NCED *transcript abundance [71]. Results from the Genevestigator response reviewer  for *AtNCED9 *(At1g78390), the Arabidopsis transcript with the highest homology with *VvNCED1*, indicates that its transcript abundance is significantly increased by ACC (the precursor of ethylene) treatment, as well as by brassinolide, heat, osmotic stress, and low concentrations of K^+^, NO_3_^- ^and glucose treatments. Thus, there are likely to be a number of factors that influence *NCED *transcript abundance.

ABA concentrations are regulated by *NCEDs *in avocado fruit, a climacteric fruit [72]. The rise in ABA concentration in avocado is preceded by a rise in ethylene concentration. Likewise, the expression of *CsNCED1 *in the peel of citrus, a non-climacteric fruit, was stimulated by ethylene and water deficit [73]. The ethylene receptor, ETR4, was found to regulate the onset of ripening in tomato fruit [74]. In a previous study, there was an increase in transcript abundance of ACC oxidase (1615952_s_at, TC56709) at stage 32 [21], which preceded the peak of *NCED1 *transcript abundance, indicating that ethylene might be a potential trigger of ABA biosynthesis at or before véraison in grape berries. However, this suggestion is very tentative as the overall correlation between the transcripts was poor and no measurements of ethylene were made. Further experiments are needed to clarify this hypothesis.

### Differential effects of water deficit on volatile compounds

Water deficit affected other metabolic pathways in berries that also act in plant defense and stress responses. These pathways involve fatty acid, isoprenoid and carotenoid metabolism as well as photosynthesis. Plant volatile esters can be derived from fatty acids through the activity of several enzymes including LOX and HPL. LOXs belong to a large gene family and are ubiquitous in plants [75]. In addition to their potential role in the formation of volatile compounds, LOXs are involved in storage lipid mobilization, plant defense and jasmonic acid biosynthesis. These enzymes might act as a "master switch" in plant development and stress adaptation [75]. HPL is a cytochrome P450 monooxygenase that is part of a small gene family, *CYP74 *[32]. This enzyme catalyzes the conversion of 13-hydroperoxide (the product of the LOX reaction) to hexanal (a green leafy volatile) and 12-oxo-cis-9-dodecenoic acid. Hexanal can be converted into other volatile compounds. These volatiles are thought to be involved in defense signaling [76]. In addition, they clearly participate in human perception of flavor and aroma in wines [77].

Water deficit had significant effects on fatty acid metabolism for both Cabernet Sauvignon and Chardonnay. Five different lipoxygenases (LOXs) were identified on the *Vitis *Genome Array with four of them having significantly increased transcript abundance in response to water deficit. However, two of these LOXs had decreased transcript abundance in Cabernet Sauvignon after véraison in response to water deficit, so the response is complex. Water deficit also increased the transcript abundance of *HPL *for both cultivars (Fig. 11).

There were specific effects of water deficit on isoprenoid and carotenoid metabolism in Chardonnay. The transcript abundance of a terpenoid synthetase and a carotenoid cleavage dioxygenase was increased by water deficit in Chardonnay at maturity, but not in Cabernet Sauvignon (Fig. 11). Both of these enzymes are known to produce odor-active volatile compounds in wine grapes [30,31]. Although these volatiles were identified in other wine grape cultivars (i.e. Muscat of Alexandria, Shiraz and Gewürztraminer) it is possible that they also contribute to the aroma profile of Chardonnay.

We did not detect significant increased concentrations of lutein and β-carotene by water deficit in our study, however a recent study [78] found small but significant increases in these carotenoids in Cabernet Sauvignon berries treated with partial rootzone drying, a specialized technique to induce water deficit and ABA concentrations. The accumulation of lutein 5,6 epoxide (Lx) in grape berries is of interest. This compound is associated with a novel long-term photoprotective/light harvesting cycle known as the Lx cycle in the leaves of a few select species, including avocado, mistletoe, shaded leaves of tropical plants and Mediterranean oaks [79,80]. Its occurrence in a fruit (avocado) has only been reported once to our knowledge [72]. Thus, this observation in grape berries presents an additional opportunity for further investigations into the physiological function of this intriguing cycle.

Glycosylated C_13_-norisoprenoid precursors in Cabernet Sauvignon berries were increased by partial rootzone drying [78]. Thus, while our data indicated that CCD transcript abundance was not increased in Cabernet Sauvignon by water deficit at maturity, earlier increased activity of this enzyme may have contributed to the increased storage (glycosylated) form. Thus, early activity of this enzyme might still contribute to perceived differences in aromas in Cabernet Sauvignon wine, because deglycosylation during yeast fermentation could convert these precursors to odor-active forms.

Aroma profiles are complex and influenced by many variables [77]. While it is not possible from this study to make direct connections between enzymatic activity and volatile production, one can link the effect of water deficit on the transcript abundance of enzymes involved in volatile production and there are clear effects of water deficit on fruity aromas in both red and white wines [36,58,5]. There were many differences between the two cultivars in response to water deficit, which might contribute to differences in their volatile and flavor profiles in the grapes and the wines. Further experiments are needed to clarify the full significance of these results.

The specific effects of water deficit on the carotenoid pathway in Chardonnay are probably related to volatile production and photoprotection. Grape berries are usually regarded as photosynthetic organs during their first phase of development, which explains why most of the transcripts with photosynthesis-related functions are largely expressed at anthesis and two weeks after flowering and then decline steadily in abundance for the remainder of berry development [21,81,82]. In grapevine shoots, water stress is known to enhance the expression of genes involved in glycolysis and the electron transport chain of chloroplasts, reflecting the need to repair damaged photosynthetic components due to photoinhibition [29]. In WD Chardonnay berries, the same response occurred with several Unigenes associated with glycolysis and photosystem II (Fig. 10). Taken together, it would appear that photosynthesis reactions were more easily damaged in WD Chardonnay berries in comparison to the anthocyanin-protected WD Cabernet Sauvignon berries.

Another fact that supports the differential occurrence of photoinhibition in Chardonnay was the altered abundance of antheraxanthin and key transcripts associated with the xanthophyll cycle. Xanthophylls protect chloroplasts from excessive light particularly under stress. For instance, chloroplasts are more tolerant of heat when they accumulate zeaxanthin [83], antheraxanthin being an intermediate step between zeaxanthin and violaxanthin. In our experiment, the transcript abundance of β-carotene hydroxylase (1615888_at, TC67433) and violaxanthin de-epoxidase (NPQ1, 1611998_at, TC68254) was increased just before maturity (Fig. 5). Conversion of violaxanthin to zeaxanthin by violaxanthin de-epoxidase is required for the dissipation of excess light energy in the antennae of photosystem II [28]. Thus, it would appear that Chardonnay berries are more sensitive during water deficit resulting in damage to the photosynthetic apparatus. To compensate for the lack of anthocyanins, white grape cultivars, like apples [46], might accumulate carotenoids to protect the photosynthetic apparatus. Anthocyanin accumulation in red or black grape berries might provide sufficient protection against excess light, which might explain why the transcript abundance of *NPQ1 *was not increased by water deficit in Cabernet Sauvignon.

In addition, flavonol concentrations were increased in Chardonnay, but not in Cabernet Sauvignon. Flavonols are phenolics; the major compounds in grapes are quercetin, kaempferol and isorhamnetin [84]. Flavonols, carotenoids and anthocyanins provide photoprotection in apples and apples with low levels of anthocyanins have increased flavonol and carotenoid concentrations [46]. Thus, the increased concentration of flavonols in WD Chardonnay berries indicates a greater need for photoprotection. An additional benefit of increased flavonol concentrations in grapes is that flavonols have important medicinal properties in humans with significant anti-inflammatory activities [85].

## Conclusion

Using transcript and metabolite profiling, water deficit was shown to have a significant impact on the metabolism of both red- and white-wine grape berries. Metabolic responses varied with the cultivar and the color of the grape. Across berry development and in response to water deficit, berry ABA concentrations were strongly correlated with the berry transcript abundance of *NCED1*. ABA concentrations were increased significantly by water deficit at véraison and one week after véraison in Cabernet Sauvignon, but not in Chardonnay berries. The rise in ABA concentrations by water deficit appeared to precede and enhance anthocyanin, hexose and proline accumulation in Cabernet Sauvignon berries. Water deficit also had large and significant impacts on the abundance of some transcripts and metabolites involved in phenylpropanoid, isoprenoid, carotenoid, amino acid and fatty acid metabolism in Cabernet Sauvignon and Chardonnay berries. To the best of our knowledge, this is the first report to show that water deficit had significant effects on transcripts involved in amino acid and fatty acid metabolism in grape berries. The effects of water deficit on metabolism have important impacts on berry constituents that influence flavor and quality characteristics in grapes and wine and might contribute to increased antioxidants and human-health benefits.

## Methods

### Experimental sites and conditions

Grape berries from Cabernet Sauvignon and Chardonnay (*Vitis vinifera*) vines were collected at seven different time points during the summer of 2004 from the Shenandoah Vineyard in Plymouth, Calfiornia, USA (Cabernet Sauvignon), and the Valley Road Field Station experimental vineyard belonging to the University of Nevada, Reno, Nevada, USA (Chardonnay). The Cabernet Sauvignon vines were 20-years-old and grown on St. George rootstock. They were grown on a T-trellis with bilateral cordons on two fruiting wires. The 8-year-old Chardonnay vines were grown on their own roots on a vertical shoot positioning trellis system with a single cordon on a single fruiting wire. To avoid any edge effects, plants used for this experiment were located in the middle of the vineyard. Initially none of the vines were irrigated in the early part of the season. Drip irrigation was initiated for the well-watered plants when stem water potentials reached -0.6 MPa and for the water-deficit vines when stem water potentials reached -1.2 MPa, the target levels for the treatments. Two grape clusters were sampled weekly on the sunny and the shady side of each plant. The clusters were pooled together in order to homogenize the samples and mitigate any potential light and temperature effects within different parts of the canopy.

### Physiological data

Six biological replicates were sampled for each measurement. Fully mature leaves were selected for stem water potentials measurements [86]. A single leaf per plant was tightly zipped in a plastic bag to eliminate transpiration at 11:00 AM in the morning; aluminum foil was wrapped around the bag, deflecting light and heat. After 120 minutes of equilibration time (1:00 PM), the excised leaf was placed into a pressure chamber (3005 Plant Water Status Console, Soil Moisture Corp., Goleta, CA, USA). The foil was removed before placing the bagged leaf in the chamber and the balancing pressure required to exude xylem sap from the cut surface was recorded. Berry development was characterized by monitoring berry diameter, total soluble solids and titratable acidity. Berry diameter was measured with a micrometer; fifteen individual berry measurements were averaged per cluster, and four clusters were sampled per vine (biological replicate). Total soluble solids (°Brix) were assayed with a refractometer (BRIX30, Leica Microsystems Inc., Buffalo, NY, USA) from the juice of crushed berries. Titratable acidity (g L^-1^) of the grape juice was assessed according to standard procedures used in the USA [87]. Statistical analyses including ANOVA analysis was performed using Prism 4.0 (GraphPad Software, Inc., San Diego, CA, USA) unless otherwise stated. Data were considered significantly different if the probability that the null hypothesis was true was p < 0.05 (i.e. means across conditions were equal).

### RNA extraction and microarray hybridization

Total RNA was extracted from berries finely ground in liquid nitrogen using Qiagen RNeasy Plant MidiKit columns as previously described [88]. The total RNA was further purified using a Qiagen RNeasy Plant Mini Kit according to the manufacturers' instructions. RNA integrity was confirmed by electrophoresis on 1.5% agarose gels containing formaldehyde and quality was confirmed by analysis on an Agilent 2100 Bioanalyzer using RNA LabChip^® ^assays according to the manufacturer's instructions. mRNAs were converted to cDNAs using a reverse transcriptase and oligo dT primer containing a T7 RNA polymerase promoter sequence. Biotinylated complementary RNAs (cRNAs) were synthesized *in vitro *using T7 RNA polymerase in the presence of biotin-labeled UTP/CTP, purified, fragmented and hybridized in the *Vitis *Genome Array cartridge (Affymetrix^®^, Santa Clara, CA, USA). The hybridized arrays were washed and stained with streptavidin phycoerythrin and biotinylated anti-streptavidin antibody using an Affymetrix Fluidics Station 400. Microarrays were scanned using a Hewlett-Packard GeneArray^® ^Scanner and image data was collected and processed on a GeneChip^® ^workstation using Affymetrix^® ^GCOS software.

### Microarray data processing

Biological triplicates were included per experimental treatment (two cultivars (Chardonnay and Cabernet Sauvignon); two irrigation levels (well-watered (WW) and water deficit (WD)); and 7 developmental stages). Expression data were processed using the same method described previously [21]. Average background and noise (RawQ) were examined for consistency across all 84 arrays. Average background ranged from 29 and 77 when run on 10% PMT scanner settings. Note that one additional array contributed a notable background level of 124.05, and without this one array, the background was mean of 49, and standard deviation of 9. RawQ levels fell between 0.7 and 3.7 with a mean value of 1.5 and standard deviation of 0.4. Present call rates were consistent across 83 or 84 arrays, ranging from 68% to 76% (mean rate 73%). One array had a call rate of 65%. This array was re-run due to inconsistencies in its RNA degradation, and upon re-running, had a present call rate of 70%. The hybridization controls BioB, BioC, BioD, and Cre were present 100% of the time. Additionally, it was verified that signal intensities of BioC, BioD, and Cre increased, respectively. Lastly, 3' to 5' ratios of both actin and GAPDH were verified to be within Affymetrix guidelines: all actin ratios were less than 1.12; GAPDH ratios were consistently below 1.33. Images of all arrays were examined, and no obvious scratches or spatial variations were observed.

Upon application of pre-processing and normalization, all 84 arrays exhibited consistent distributions (Additional files 6 and 7). Similarly, digestion curves describing trends in RNA degradation between the 5' end and the 3' end of each probe set were generated, and all 84 proved comparable (Additional file 8).

To ensure strict reproducibility standards, any probe set in which less than two of the triplicates were not present (as labeled by the Affymetrix GCOS software) was deleted. There were 6,501 probe sets (39%) that did not pass this rigorous control measure, and were deleted.

A simple, three-way fixed effects ANOVA was performed on the RMA-normalized (Robust Multichip Average; [89]) and processed data to examine probe sets with significant Treatment effects, Treatment and Cultivar interaction effects, and Treatment, Cultivar, and Time interaction effects. A multiple testing correction was applied to the *p*-values of the F-statistics to adjust the false discovery rate [90]. Genes with adjusted F-statistic *p*-values < 0.05 were extracted for further analysis. The raw and processed data have been deposited in PLEXdb (; experiment: VV5).

### qRT-PCR experiments

RNA was extracted from the sample biological sample as for hybridization of the *Vitis *Genome Array and its integrity was verified as described above. cDNA was synthesized using an iScript cDNA Synthesis Kit (Bio-Rad Laboratories, Hercules, CA, USA) according to manufacturers' instructions with a uniform 1 μg RNA per reaction volume reverse-transcribed. Primers for genes (Additional file 1) assayed by quantitative real-time RT-PCR (qRT-PCR) were selected using Primer3 software [91]. qRT-PCR reactions were prepared using an iTaq SYBR Green Supermix with ROX (Bio-Rad) and performed using the ABI PRISM^® ^7000 Sequence Detection System (Applied Biosystems, Foster City, CA, USA). Expression was determined for triplicate biological replicates by use of serial dilution cDNA standard curves per gene. In order to assess the performance of the array in a biological context, we examined the transcript abundance of some candidate genes from Cabernet Sauvignon and Chardonnay exhibiting a marked expression pattern across the seven time points in WW and WD conditions. qRT-PCR was performed with the ABI PRISM 7000 Sequence Detection System (Applied Biosystems) under annealing conditions of 50°C for 1 minute and analyzed with ABI PRISM 700 SDS software. Analysis of relative gene expression was performed using the  method [92]. Comparative analyses between microarray data and real time gene expression were analyzed using the equation ΔΔ C_*T *_= (C_T, Target _- C_T, HG_) Time_*X *_- (C_T, Target _- C_T, HG_) Time_0 _where Time_*X *_is the value at any time point and Time_0 _represents the 1× expression of the target gene normalized to the housekeeping gene (HG). Specific qRT-PCR for *NCED1 *and *NCED2 *were carried out by comparison of the Ct data between the target gene and the housekeeping gene throughout berry development. Primer pairs used for the relative gene expression of *NCED1 *and *NCED2 *isogenes were the same as those described previously [23]. Data were calculated from the calibration curve and normalized [93] using the expression curve of an ankyrin-repeat protein (1612584_s_at; TC53110), whose mRNA presented an extremely low coefficient of variation (0.056, M Value = 0.1297) with microarray analysis.

### Metabolite extraction and derivatization protocol of the polar extracts of berry

All tissue samples were kept frozen throughout the freeze-drying procedure. Freeze-dried berry tissue (6 mg) was placed in a standard screw-cap-threaded, glass vial. Polar metabolites were extracted with a water/chloroform protocol according to previously established procedures [94]. The aqueous phase, after 1 hour of extraction, containing 12.5 mg L^-1 ^of ribitol as an internal standard, was evaporated over-night in a vacuum concentrator and the tube was then returned to the -80°C freezer until use. Afterwards, polar samples were derivatized by adding 120 μL of 15 mg mL^-1 ^of methoxyamine HCl in pyridine, sonicated until all crystals disappeared and incubated at 50°C for 30 minutes. After that, 120 μL of MSTFA + 1% TMCS (Sigma-Aldrich, Inc., St. Louis, MO, USA) were added, incubated at 50°C for 30 minutes and immediately taken for analysis with a Thermo Finnigan Polaris Q230 GC-MS (Thermo Electron Corporation, Waltham, MA, USA). Derivatized samples (120 μL) were transferred to a 200 μL silanized vial insert and run at an injection split of 200:1 and 10:1 to bring the large and weak peaks to a concentration within the range of the detector. The inlet and transfer lines were held at 240°C and 320°C, respectively. Separation was achieved with a temperature program of 80°C for 3 min, then ramped at 5°C min^-1 ^to 315°C and held for 17 min, using a 60-m DB-5MS column (J&W Scientific, 0.25 mm ID, 0.25 μm film thickness) and a constant flow of 1.0 ml min^-1^. All organic acids, sugars and amino acids were verified with standards purchased from Sigma-Aldrich.

### Metabolite data processing

Metabolites were identified in the chromatograms using one software package: Xcalibur (1.3; Thermo Electron Corporation). The software matched the mass spectrum in each peak against three different metabolite libraries: NIST (v2.0: ), Golm (T_MSRI_ID: ) and our own custom-created UNR library (V1) made from standards bought from Sigma-Aldrich. Quantification of the area of the chromatogram peaks was determined using Xcalibur and normalized as a ratio of the area of the compound peak to the area of the ribitol internal standard. ANOVA and other statistical analyses were performed using Prism 4.0 (GraphPad Software, Inc, USA).

### Extraction and quantification of anthocyanins

The extraction and quantification of anthocyanins followed methods described previously [95]. Freeze-dried cells were extracted with 1% trifluoroacetic acid (TFA) in methanol (MeOH). The filtered extracts were combined and concentrated in a vacuum concentrator. The obtained aqueous solution was applied to an Amberlite XAD-7 chromatographic column [96] and washed with 1% aqueous TFA. Anthocyanins were eluted by 50% MeOH containing 1% aqueous TFA. In the next step of purification, the crude anthocyanins were separated by semi-preparative HPLC on a Protonsil Eurobond (Bischof, Germany) reverse-phase C18 column (5 μm packing, 4 mm i.d. × 250 mm) protected with a guard column of the same material. Solvents used for the separation were 0.1% trifluoroacetic acid in water (A) and 0.1% trifluoroacetic acid in acetonitrile (B). The elution program at 0.6 mL/min was 12% to 26% B (0–35 min), 26% to 100% B (35–36 min), 100% B (36–49 min), 100% to 12% B (49–50 min) and 12% B (50–60 min). The chromatogram was monitored at 521 nm. HPLC analyses were carried out on a Gilson (Middleton, Wisconsin, USA) gradient system (305/306 pumps driven by 712 system controller software) equipped with a UV visible detector (Model SP8450, Spectra Physics, Inc, Irvine, California, USA). Delphinidin- and petunidin-3-O-β-glucosides were collected at retention times (Rt) of 17 min and 21 min, respectively. Cyanidin-, peonidin- and malvidin-3-O-β-glucosides were collected at RT of 19 min, 24 min and 25 min. Their contents were estimated from calibration curves established with pure compounds purchased from Extrasynthèse (Genay Cedex, France). Their chemical structures were controlled by their mass (in the Fast-Atom bombardment^+ ^mode) and their H^1^-NMR spectra (data not shown) [97].

### Extraction and determination of flavonols

Flavonols were determined with Neu's reagent solution [98]. A 200 μL aliquot of berry extract was mixed with 900 μL of the reagent solution (50 μL of 2-aminoethyl diphenylborinate (Fluka, Inc., Buchs, Switzerland) solution in 1% of methanol + 850 μL of methanol). The absorbance of the solution was measured at 410 nm and the flavonols were expressed as mg rutin (Extrasynthèse) equivalent.

### Extraction and determination of chlorophyll

Frozen berry tissue (0.2 g) was ground in 1 mL of extraction buffer (100 mM Tris pH = 7.8, 10 mM MgCl_2_, 10 mM DTT, 1% PVP-360, 1% Triton X-100). The samples were centrifuged for 10 minutes at 4°C. A 200 μL aliquot was resuspended in 800 μL of 100% acetone for the chlorophyll determination. The sample was then centrifuged at 15,000 rpm for 10 minutes at 4°C. Chlorophyll was assayed in a spectrophotometer according to Guralnick and Ting [99].

### Extraction and determination of ABA and its metabolites

ABA extraction was based on a method published previously [100,101] with some modifications due to the presence of phenolic compounds. An internal, deuterated standard mixture (IS mix) was prepared containing (+)- 4,5,8',8',8'-d5-ABA-GE and (-)-5,8',8',8'-d_4 _ABA (Plant Biotechnology Institute, Saskatoon, Canada). The non-labeled mixture included (+)-ABA-GE and (±)-7'OH-ABA and was used to produce quality control (QC) standards, which were prepared and analyzed along with each batch of samples. Both mixtures were prepared in water: acetonitrile: glacial acetic acid (H_2_O:ACN:AcOH) (50:49.5:0.5), containing equal amounts of each standard (3 ng μL^-1^). The reconstitution mix was dissolved in the starting solvent of the HPLC gradient (H_2_O:ACN, 85:15, with 0.1% AcOH).

Aliquots of 50 to 100 mg of lyophilized whole grape berry samples were weighed out into a 6 mL screw-cap centrifuge tube, to which was added 3 mL of extraction solvent of isopropyl alcohol:H_2_O:AcOH (80:19:1) along with a 60 ng equivalent of each of the deuterium-labeled standards. After vortexing for one min, the solution was put onto an orbital shaker that was placed in a refrigerator at ~4°C, and the solution allowed to extract for 24 hours. After extraction, the samples were centrifuged for 20 minutes at 2500 × g and the supernatant removed from the centrifuge tube and transferred to a glass test tube (2 mL screw thread). The solids left in the centrifuge tube were washed with another 0.5 mL portion of the extraction solvent, vortexed and centrifuged again, and the supernatant added to the first portion removed from the vial. The supernatant was dried down in a centrifugal concentrator (GMI, Ramsey, USA) at room temperature and 10 × g.

The dried extract was reconstituted in 2 mL of H_2_0:AcOH (99:1) for cleanup by mixed-mode cation exchange solid-phase extraction (Oasis MCX SPE cartridges, 3 cc, 60 mg, Waters, Milford, MA, USA). The cartridges were first prepared by running 3 mL MeOH:AcOH (99:1) and then 3 mL H_2_O:AcOH (99:1) through them into waste vials in the vacuum manifold to which they were attached. Samples were then added to each cartridge and allowed to run through at a rate of approximately one drop every 1 to 2 seconds. The sample vials were each washed with 0.5 mL H_2_O:AcOH (99:1) and this wash was added to the appropriate cartridge as they were draining. Next, each cartridge was washed with 1 mL H_2_O:AcOH (99:1), which also dripped through at the same rate. The cartridges were eluted after centrifugation at 45 × g for 1 min into 1.5 mL of MeOH:AcOH (99:1) by adapting the cartridges onto a microcentrifuge. Finally, the eluate was dried down in a centrifugal concentrator at room temperature.

The dried extracts were re-dissolved in 100 μL MeOH:AcOH (99:1) and made up to 1 mL in H_2_O:AcOH (99:1) for a second stage of solid phase extraction using hydrophilic-lipophilic balance cartridges (Oasis HLB, 1 cc, 30 mg, Waters, Milford, MA, USA). The cartridges were first prepared by washing and equilibrating with 1 mL MeOH:AcOH (99:1) and 1 mL H_2_O:AcOH (99:1), respectively, before loading the sample. Again, the drip rate was kept to approximately one drop every 1 to 2 seconds, and a second wash of the microcentrifuge tube, using 0.5 mL H_2_O:AcOH (99:1), was added as the sample was drawn through the cartridge. The sample was washed with 1 mL H_2_O:AcOH (99:1) and finally eluted into a second microcentrifuge tube using 1 mL H_2_O:CAN:AcOH (69:30:1). This final extract was dried down in a centrifugal concentrator (GMI) and re-dissolved in 80 μL of the reconstitution mix prior to LC-MS/MS analysis.

All samples were analyzed using a Michrom Paradigm MDLC (Michrom Bioresources Inc, Auburn, California, USA) coupled to a LCQ DECA XP+ ion trap mass spectrometer (Thermo Finnigan, San Jose, California, USA). The mobile phase solvent composition and gradient is listed in Table 1. A flow rate of 200 μL min^-1 ^using a Symmetry C18 column (2.1 × 100 mm, 3.5 um, Waters, Milford, Massachusetts, USA) and auto-injection of 20 μL using a Paradigm AS1 (Michrom Bioresources Inc., Auburn, California, USA). The electrospray mass spectrometer was operated under negative ion mode using a needle potential of 2.50 KV with a capillary temperature of 220°C and a sheath flow of 20. The scan ranges and conditions for ABA-GE and ABA are listed in Table 2. Because only the deuterated ABA internal standard was adequately detected, standard curves of all ABA metabolites were subsequently generated against the ABA internal standard in order to calculate their concentrations in the berries.

**Table 1:** Mobile phase solvent composition and gradient protocol.

**Time (min)**	**Percent** **Solvent A**	**Percent** **Solvent B**	**Percent** **Solvent C**
0.00	94.00	5.00	1.00
30.00	64.00	35.00	1.00
34.20	59.00	40.00	1.00
35.00	39.00	60.00	1.00
38.20	39.00	60.00	1.00
38.30	0.00	99.00	1.00
39.50	0.00	99.00	1.00
40.50	94.00	5.00	1.00
50.00	94.00	5.00	1.00

Solvent A is water, Solvent B is acetonitrile, and Solvent C is 5% formic acid.

**Table 2:** LC-MS conditions for ABA and its metabolites.

**Analyte**	**Precursor m/z**	**Product m/z**	**CE %**	**Retention Time**
DPA	281.1	171.1	35	0.0 – 15.00
d_3_-DPA	284.1	174.1	35	0.0 – 15.00
ABA-GE	425.1	263.1	40	15.00 – 18.25
d_5_-ABA-GE	430.1	268.1	40	15.00 – 18.25
PA	279.1	139.1	35	18.25 – 21.00
d_3_-PA	282.1	142.1	35	18.25 – 21.00
ABA	263.1	153.1	35	21.00 – 50.00
d_4_-ABA	267.1	156.1	35	21.00 – 50.00

### Extraction and determination of carotenoids

Carotenoids were extracted from grape berries as described previously [102] with some modifications. Aliquots of 200 mg of finely crushed frozen berries (liquid N_2_) were homogenized in 600 μL of distilled-deionized water and 1 μg of internal standard (β-apo-8'-carotenal) was added to assess the recovery of carotenoids after the extraction. Butylated hydroxytoluene (200 mg per sample) was added as a preservative to protect the samples from oxidation. Extraction was carried out with 600 μL of HPLC grade ether/hexane (1:1, v/v) and agitated for 30 min. The extraction was repeated two more times. The three different upper phases obtained were combined and concentrated to dryness in a speedVac. The extract was resuspended in 50 μL acetone/hexane (1:1, v/v) for HPLC determination. Light exposure was minimized during sample preparations in order to avoid photoisomerization.

Carotenoids were analyzed using an Agilent 1100 series HPLC (Agilent Technologies, Inc, Santa Clara, California, USA) equipped with Chemstation software (v. 10.02), a UV-visible photodiode array detector and a Spherisorb^® ^ODS2 (5 μm particle size; 250 mm × 4.6 mm ID) C18 column (Waters, Milford, MA, USA). The absorption spectra were recorded from 270 to 550 nm. The HPLC conditions for the mobile phase were solvent A, ethyl acetate (HPLC grade); solvent B, acetonitrile/water (9:1, v/v); flow rate = 1 mL min^-1^. The following gradient was employed, 0 to 31 min (0 to 60%A); 31 to 46 min (60%A); 46 to 51 min (60 to 100%A); 51 to 55 min (100%A); 55 to 60 min (100 to 0% A); 60 to 65 min (0% A). Carotenoids were identified by comparison with their absorption spectra [26], commercially available standards (β-carotene and lutein, Sigma-Aldrich, Inc.) and purified compounds from Arabidopsis and avocado leaves [79,103]. Amounts were quantified relative to the internal standard, β-apo-8'-carotenal. An example chromatogram and absorption spectra are presented in Additional file 9.

## Authors' contributions

LGD performed the mRNA extraction, qRT-PCR, all metabolite analyses including GC-MS analyses and HPLC analyses, analyzed the microarray experiments, prepared figures and tables and wrote the initial manuscript draft. MDW and GRC acquired physiological data. JG assisted in data analysis and transcript annotation. DQ supervised the GC-MS analysis. KAS performed the quantitative and statistical analyses for the microarrays. AD and JMM performed the anthocyanin analyses. JCC participated in the organization of the study, preparation and finalization of the manuscript. GRC organized the study, assisted in the carotenoid analyses, annotated and functionally categorized transcripts, and finalized the written manuscript.

## Supplementary Material

Additional file 1 **Forward and reverse primers used with qRT-PCR**. This file provides a list of all of the forward and reverse primers used with qRT-PCR for the estimation of relative transcript abundance. Click here for file

Additional file 2 **Linear regression of the transcript abundance values of the qRT-PCR with the corresponding values of the same transcript on the microarray**. qRT-PCR data are on the y-axis and the microarray data are on the x-axis. Each value represents the mean of 6 different time points for each transcript. Data were normalized to the transcript abundance of an ankyrin-repeat protein (1612584_s_at) that did not change with developmental stage or stress treatment. Ferulate-5-hydroxylase (F5H, 1614502_at, TC63764) is represented by solid grey squares (Cabernet Sauvignon with well-watered conditions; CS-WW) and by red triangles (Cabernet Sauvignon with water deficit; CS-WD). 9-cis-epoxycarotenoid dioxygenase (NCED1, 1608022_at, TC57089) is represented by orange triangles (CS-WW) and orange diamonds (CS-WD). Expansin (1607674_at, TC54149) is represented by solid green circles (Chardonnay with well-watered conditions; CH-WW) and open light blue triangles (CH-WD). Diacyl glycerol kinase 2 (1618308_at, TC63661) is represented by open green squares (CH-WW) and open dark blue triangles (CH-WD). Click here for file

Additional file 3 **Probe sets that exhibited a significant Treatment effect in their expression levels**. The list of probe sets was selected based upon statistical analysis. Click here for file

Additional file 4 **Probe sets that exhibited a significant Cultivar and Treatment effect**. The list of probe sets was selected based upon statistical analysis. Click here for file

Additional file 5 **Probe sets that exhibited a significant Cultivar, Treatment, and Time interaction effect**. The list of probe sets was selected based upon statistical analysis. Click here for file

Additional file 6 **Post-RMA Perfect Match (PM) intensity boxplots of all 84 *Vitis *Genome Arrays**. This is a plot to assess the quality of the data from the arrays. Click here for file

Additional file 7 **Post-RMA Perfect Match (PM) intensity distributions of all 84 *Vitis *Genome Arrays**. This is a plot to assess the quality of the data from the arrays. Click here for file

Additional file 8 **RNA digestion plot of all 84 *Vitis *Genome Arrays**. This is a plot to assess the quality of the data from the arrays. Click here for file

Additional file 9 **A representative HPLC chromatogram for carotenoid identification and quantification**. This is a plot to assess the quality of the data and the retention times from the chromatogram. Click here for file

## References

[B1] McKersie BD, Leshem Y (1994). Stress and stress coping in cultivated plants.

[B2] Grimplet J, Deluc LG, Cramer GR, Cushman JC, Jenks MA, Hasegawa PM, Jain SM (2007). Integrating functional genomics with abiotic stress responses in wine grape – *Vitis vinifera*. Advances in Molecular Breeding towards Salinity and Drought Tolerance.

[B3] Hardie WJ, Considine JA (1976). Response of grapes to water-deficit stress in particular stages of development. Am J Enol Vitic.

[B4] Matthews MA (1989). Reproductive development in grape (*Vitis vinifera *L.): responses to seasonal water deficits. Am J Enol Vitic.

[B5] Chapman DM, Roby G, Ebeler SE, Guinard J-X, Matthews MA (2005). Sensory attributes of Cabernet Sauvignon wines made from vines with different water status. Aust J Grape Wine Res.

[B6] Roby G, Harbertson JF, Adams DA, Matthews MA (2004). Berry size and vine water deficits as factors in winegrape composition: anthocyanins and tannins. Aust J Grape Wine Res.

[B7] Matthews MA, Anderson MM (1988). Fruit ripening in *Vitis vinifera *L.: responses to seasonal water deficits. Am J Enol Vitic.

[B8] Matthews MA, Ishii R, Anderson MM, O'Mahony M (1990). Dependence of wine sensory attributes on vine water status. J Sci Food Agric.

[B9] Castellarin SD, Matthews MA, Di Gaspero G, Gambetta GA (2007). Water deficits accelerate ripening and induce changes in gene expression regulating flavonoid biosynthesis in grape berries. Planta.

[B10] Ojeda H, Andary C, Kraeva E, Carbonneau A, Deloire A (2002). Influence of pre- and postveraison water deficit on synthesis and concentration of skin phenolic compounds during berry growth of *Vitis vinifera *cv. Shiraz. Am J Enol Vitic.

[B11] Geny L, Saucier C, Bracco S, Daviaud F, Glories Y (2003). Composition and cellular localization of tannins in grape seeds during maturation. J Agric Food Chem.

[B12] Neja RA, Wildman WE, Ayers RS, Kasimatis AN (1977). Grapevine response to irrigation and trellis treatments in the Salinas Valley. Am J Enol Vitic.

[B13] Smart R, Coombe B (1983). Water relations of grapevines.

[B14] Grimplet J, Deluc LG, Tillett RL, Wheatley MD, Schlauch KA, Cramer GR, Cushman JC (2007). Tissue-specific mRNA expression profiling in grape berry tissues. BMC Genomics.

[B15] Castellarin SD, Pfeiffer A, Sivilotti P, Degan M, Peterlunger E, DIG G (2007). Transcriptional regulation of anthocyanin biosynthesis in ripening fruits of grapevine under seasonal water deficit. Plant Cell Environ.

[B16] Hiratsuka S, Onodera H, Kawai Y, Kubo T, Itoh H, Wada R (2001). ABA and sugar effects on anthocyanin formation in grape berry cultured *in vitro*. Sci Hort.

[B17] Soar CJ, Speirs J, Maffei SM, Loveys BR (2004). Gradients in stomatal conductance, xylem sap ABA and bulk leaf ABA along canes of *Vitis vinifera *cv. Shiraz: molecular and physiological studies investigating their source. Funct Plant Biol.

[B18] Okamoto G, Kuwamura T, Hirano K (2004). Effects of water deficit stress on leaf and berry ABA and berry ripening in Chardonnay grapevines (*Vitis vinifera*). Vitis.

[B19] Pou A, Flexas J, Alsina Mdel M, Bota J, Carambula C, de Herralde F, Galmes J, Lovisolo C, Jimenez M, Ribas-Carbo M (2008). Adjustments of water use efficiency by stomatal regulation during drought and recovery in the drought-adapted Vitis hybrid Richter-110 (V. berlandieri × V. rupestris). Physiol Plant.

[B20] Coombe BG (1992). Research on development and ripening of the grape berry. Am J Enol Vitic.

[B21] Deluc LG, Grimplet J, Wheatley MD, Tillett RL, Quilici DR, Osborne C, Schooley DA, Schlauch KA, Cushman JC, Cramer GR (2007). Transcriptomic and metabolite analyses of Cabernet Sauvignon grape berry development. BMC Genomics.

[B22] Schoof H, Ernst R, Nazarov V, Pfeifer L, Mewes HW, Mayer KF (2004). MIPS *Arabidopsis thaliana *Database (MAtDB): an integrated biological knowledge resource for plant genomics. Nucleic Acids Res.

[B23] Lund ST, Peng FY, Nayar T, Reid KE, Schlosser J (2008). Gene expression analyses in individual grape (*Vitis vinifera *L.) berries during ripening initiation reveal that pigmentation intensity is a valid indicator of developmental staging within the cluster. Plant Mol Biol.

[B24] Söderman E, Mattsson J, Engström P (1996). The *Arabidopsis *homeobox gene *ATHB-7 *is induced by water deficit and by abscisic acid. Plant J.

[B25] Olsson AS, Engstrom P, Soderman E (2004). The homeobox genes ATHB12 and ATHB7 encode potential regulators of growth in response to water deficit in Arabidopsis. Plant Mol Biol.

[B26] Cuttriss AJ, Mimica JL, Pogson BJ, Howitt CA, Wise RR, Hoober JK (2006). Carotenoids. The Structure and Function of Plastids.

[B27] Fang J, Chai C, Qian Q, Li C, Tang J, Sun L, Huang Z, Guo X, Sun C, Liu M (2008). Mutations of genes in synthesis of the carotenoid precursors of ABA lead to pre-harvest sprouting and photo-oxidation in rice. Plant J.

[B28] Horton P, Wentworth M, Ruban A (2005). Control of the light harvesting function of chloroplast membranes: the LHCII-aggregation model for non-photochemical quenching. FEBS Lett.

[B29] Cramer GR, Ergul A, Grimplet J, Tillett RL, Tattersall EA, Bohlman MC, Vincent D, Sonderegger J, Evans J, Osborne C (2007). Water and salinity stress in grapevines: early and late changes in transcript and metabolite profiles. Funct Integr Genomics.

[B30] Lücker J, Bowen P, Bohlman J (2004). *Vitis vinifera *terpenoid cyclases: functional identification of two sesquiterpene synthase cDNAs encoding (+)-valencene synthase and (-)-germacrene D synthase and expression of mono- and sesquiterpene synthases in grapevine flowers and berries. Phytochemistry.

[B31] Mathieu S, Terrier N, Procureur J, Bigey F, Gunata Z (2005). A carotenoid cleavage dioxygenase from *Vitis vinifera *L.: functional characterization and expression during grape berry development in relation to C13-norisoprenoid accumulation. J Exp Bot.

[B32] Duan H, Huang MY, Palacio K, Schuler MA (2005). Variations in CYP74B2 (hydroperoxide lyase) gene expression differentially affect hexenal signaling in the Columbia and Landsberg erecta ecotypes of *Arabidopsis*. Plant Physiol.

[B33] Feussner I, Wasternack C (2002). The lipoxygenase pathway. Annu Rev Plant Biol.

[B34] Speirs J, Lee E, Holt K, Yong-Duk K, Scott NS, Loveys B, Schuch W (1998). Genetic manipulation of alcohol dehydrogenase levels in ripening tomato fruit affects the balance of some flavor aldehydes and alcohols. Plant Physiol.

[B35] Tesniere C, Torregrosa L, Pradal M, Souquet JM, Gilles C, Dos Santos K, Chatelet P, Gunata Z (2006). Effects of genetic manipulation of alcohol dehydrogenase levels on the response to stress and the synthesis of secondary metabolites in grapevine leaves. J Exp Bot.

[B36] Cramer GR, Evans J, Ardelean R, Keady M, Quilici D, Schooley DA, Jeandet P, Clément C, Conreaux A (2007). Impacts of regulated-deficit irrigation on the flavor components of grapes and wines. Macromolecules and Secondary Metabolites of Grapevine and Wine.

[B37] Coruzzi G, Last R, Buchanan B, Gruissem W, Jones R (2000). Amino Acids. Biochemistry and Molecular Biology of Plants.

[B38] Forde BG, Lea PJ (2007). Glutamate in plants: metabolism, regulation, and signalling. J Exp Bot.

[B39] Cheynier V, Duenas-Paton M, Salas E, Maury C, Souquet J-M, Sarni-Manchado P, Fulcrand H (2006). Structure and properties of wine pigments and tannins. Am J Enol Vitic.

[B40] Pezzuto JM (2008). Grapes and Human Health: A Perspective. J Agric Food Chem.

[B41] Aron PM, Kennedy JA (2008). Flavan-3-ols: nature, occurrence and biological activity. Mol Nutr Food Res.

[B42] Szajdek A, Borowska EJ (2008). Bioactive Compounds and Health-Promoting Properties of Berry Fruits: A Review. Plant Foods Hum Nutr.

[B43] Kobayashi S, Goto-Yamamoto N, Hirochika H (2004). Retrotransposon-induced mutations in grape skin color. Science.

[B44] Kobayashi S, Ishimaru M, Hiraoka K, Honda C (2002). Myb-related genes of the Kyoho grape (*Vitis labruscana*) regulate anthocyanin biosynthesis. Planta.

[B45] Walker AR, Lee E, Bogs J, McDavid DA, Thomas MR, Robinson SP (2007). White grapes arose through the mutation of two similar and adjacent regulatory genes. Plant J.

[B46] Merzlyak MN, Melo TB, Naqvi KR (2008). Effect of anthocyanins, carotenoids, and flavonols on chlorophyll fluorescence excitation spectra in apple fruit: signature analysis, assessment, modelling, and relevance to photoprotection. J Exp Bot.

[B47] Pereira GE, Gaudillere JP, Pieri P, Hilbert G, Maucourt M, Deborde C, Moing A, Rolin D (2006). Microclimate influence on mineral and metabolic profiles of grape berries. J Agric Food Chem.

[B48] Fujita A, Goto-Yamamoto N, Aramaki I, Hashizume K (2006). Organ-specific transcription of putative flavonol synthase genes of grapevine and effects of plant hormones and shading on flavonol biosynthesis in grape berry skins. Biosci Biotechnol Biochem.

[B49] Verhoeyen ME, Bovy A, Collins G, Muir S, Robinson S, de Vos CH, Colliver S (2002). Increasing antioxidant levels in tomatoes through modification of the flavonoid biosynthetic pathway. J Exp Bot.

[B50] Bowers JE, Meredith CP (1997). The parentage of a classic wine grape, Cabernet Sauvignon. Nat Genet.

[B51] Bowers J, Boursiquot JM, This P, Chu K, Johansson H, Meredith C (1999). Historical genetics: The parentage of Chardonnay, Gamay, and other wine grapes of northeastern France. Science.

[B52] Vincent D, Ergul A, Bohlman MC, Tattersall EA, Tillett RL, Wheatley MD, Woolsey R, Quilici DR, Joets J, Schlauch K (2007). Proteomic analysis reveals differences between Vitis vinifera L. cv. Chardonnay and cv. Cabernet Sauvignon and their responses to water deficit and salinity. J Exp Bot.

[B53] Tattersall EA, Grimplet J, Deluc L, Wheatley MD, Vincent D, Osborne C, Ergul A, Lomen E, Blank RR, Schlauch KA (2007). Transcript abundance profiles reveal larger and more complex responses of grapevine to chilling compared to osmotic and salinity stress. Funct Integr Genomics.

[B54] Ribaut JM, Pilet PE (1991). Effects of water stress on growth, osmotic potential and abscisic acid content of maize roots. Physiol Plant.

[B55] He T, Cramer GR (1996). Abscisic acid concentrations are correlated with leaf area reductions in two salt-stressed rapid-cycling *Brassica *species. Plant Soil.

[B56] Munns R, Cramer GR (1996). Is coordination of leaf and root growth mediated by abscisic acid? Opinion. Plant Soil.

[B57] Nambara E, Marion-Poll A (2005). Abscisic acid biosynthesis and catabolism. Annu Rev Plant Biol.

[B58] Koundouras S, Marinos V, Gkoulioti A, Kotseridis Y, van Leeuwen C (2006). Influence of vineyard location and vine water status on fruit maturation of nonirrigated cv. Agiorgitiko (*Vitis vinifera *L.). Effects on wine phenolic and aroma components. J Agric Food Chem.

[B59] Conde C, Agasse A, Glissant D, Tavares R, Geros H, Delrot S (2006). Pathways of glucose regulation of monosaccharide transport in grape cells. Plant Physiol.

[B60] Cakir B, Agasse A, Gaillard C, Saumonneau A, Delrot S, Atanassova R (2003). A grape ASR protein involved in sugar and abscisic acid signaling. Plant Cell.

[B61] Leterrier M (2002). Régulation et rôle physiologique du gène VvHT1 exprimé durant la maturation de la baie de raisin.

[B62] Urano K, Maruyama K, Ogata Y, Morishita Y, Takeda M, Sakurai N, Suzuki H, Saito K, Shibata D, Kobayashi M (2009). Characterization of the ABA-regulated global responses to dehydration in *Arabidopsis *by metabolomics. Plant J.

[B63] Pan Q-H, Li M-J, Peng C-C, Zhang N, Zou X, Zou K-Q, Wang X-L, Yu X-C, Wang X-F, Zhang D-P (2005). Abscisic acid activates acid invertases in developing grape berry. Physiol Plant.

[B64] Schwartz SH, Qin X, Zeevaart JA (2003). Elucidation of the indirect pathway of abscisic acid biosynthesis by mutants, genes, and enzymes. Plant Physiol.

[B65] Iuchi S, Kobayashi M, Taji T, Naramoto M, Seki M, Kato T, Tabata S, Kakubari Y, Yamaguchi-Shinozaki K, Shinozaki K (2001). Regulation of drought tolerance by gene manipulation of 9-cis-epoxycarotenoid dioxygenase, a key enzyme in abscisic acid biosynthesis in *Arabidopsis*. Plant J.

[B66] Thomas TR, Matthews MA, Shackel KA (2006). Direct *in situ *measurement of cell turgor in grape (*Vitis vinifera *L.) berries during development and in response to plant water deficits. Plant Cell Environ.

[B67] Wada H, Shackel KA, Matthews MA (2008). Fruit ripening in *Vitis vinifera*: apoplastic solute accumulation accounts for pre-veraison turgor loss in berries. Planta.

[B68] Zhang XY, Wang XL, Wang XF, Xia GH, Pan QH, Fan RC, Wu FQ, Yu XC, Zhang DP (2006). A shift of phloem unloading from symplasmic to apoplasmic pathway is involved in developmental onset of ripening in grape berry. Plant Physiol.

[B69] Hansen H, Grossmann K (2000). Auxin-induced ethylene triggers abscisic acid biosynthesis and growth inhibition. Plant Physiol.

[B70] Weatherwax SC, Ong MS, Degenhardt J, Bray EA, Tobin EM (1996). The interaction of light and abscisic acid in the regulation of plant gene expression. Plant Physiol.

[B71] Sawada Y, Aoki M, Nakaminami K, Mitsuhashi W, Tatematsu K, Kushiro T, Koshiba T, Kamiya Y, Inoue Y, Nambara E (2008). Phytochrome- and gibberellin-mediated regulation of abscisic acid metabolism during germination of photoblastic lettuce seeds. Plant Physiol.

[B72] Chernys JT, Zeevaart JA (2000). Characterization of the 9-cis-epoxycarotenoid dioxygenase gene family and the regulation of abscisic acid biosynthesis in avocado. Plant Physiol.

[B73] Rodrigo MJ, Alquezar B, Zacarias L (2006). Cloning and characterization of two 9-cis-epoxycarotenoid dioxygenase genes, differentially regulated during fruit maturation and under stress conditions, from orange (*Citrus sinensis *L. Osbeck). J Exp Bot.

[B74] Kevany BM, Taylor MG, Klee HJ (2008). Fruit-specific suppression of the ethylene receptor LeETR4 results in early-ripening tomato fruit. Plant Biotechnol J.

[B75] Liavonchanka A, Feussner I (2006). Lipoxygenases: Occurrence, functions and catalysis. J Plant Physiol.

[B76] Halitschke R, Ziegler J, Keinanen M, Baldwin IT (2004). Silencing of hydroperoxide lyase and allene oxide synthase reveals substrate and defense signaling crosstalk in *Nicotiana attenuata*. Plant J.

[B77] Lee SJ, Noble AC (2003). Characterization of odor-active compounds in Californian Chardonnay wines using GC-olfactometry and GC-mass spectrometry. J Agric Food Chem.

[B78] Bindon KA, Dry PR, Loveys BR (2007). Influence of plant water status on the production of C13-norisoprenoid precursors in Vitis vinifera L. Cv. cabernet sauvignon grape berries. J Agric Food Chem.

[B79] Förster B, Osmond CB, Pogson BJ (2009). De-novo synthesis and degradation of Lx- and V-cycle pigments during shade and sun acclimation in avocado leaves (*P. americana*). Plant Physiol.

[B80] García-Plazaola JI, Matsubara S, Osmond CB (2007). The lutein epoxide cycle in higher plants: its relationships to other xanthophyll cycles and possible functions. Funct Plant Biol.

[B81] Terrier N, Glissant D, Grimplet J, Barrieu F, Abbal P, Couture C, Ageorges A, Atanassova R, Leon C, Renaudin JP (2005). Isogene specific oligo arrays reveal multifaceted changes in gene expression during grape berry (*Vitis vinifera *L.) development. Planta.

[B82] Waters DL, Holton TA, Ablett EM, Lee LS, Henry RJ (2005). cDNA microarray analysis of developing grape (*Vitis vinifera *cv. Shiraz) berry skin. Funct Integr Genomics.

[B83] Havaux M, Tardy F, Ravenel J, Chanu D, Parot P (1996). Thylakoid membrane stability to heat stress studied by flash spectroscopic measurements of the electrochromic shift in intact potato leaves: influence of the xanthophyll content. Plant Cell Environ.

[B84] Mattivi F, Guzzon R, Vrhovsek U, Stefanini M, Velasco R (2006). Metabolite profiling of grape: flavonols and anthocyanins. J Agric Food Chem.

[B85] Graf BA, Milbury PE, Blumberg JB (2005). Flavonols, flavones, flavanones, and human health: epidemiological evidence. J Med Food.

[B86] McCutchan J, Shackel KA (1992). Stem-water potential as a sensitive indicator of water stress in prune trees (*Prunus domestica *L. cv. French). J Amer Soc Hort Sci.

[B87] Guymon JF, Ough CS (1962). A uniform method for total acid determination in wines. Am J Enol Vitic.

[B88] Tattersall EAR, Ergul A, AlKayal F, Deluc L, Cushman JC, Cramer GR (2005). Comparison of methods for isolating high-quality RNA from leaves of grapevine. Am J Enol Vitic.

[B89] Irizarry RA, Hobbs B, Collin F, Beazer-Barclay YD, Antonellis KJ, Scherf U, Speed TP (2003). Exploration, normalization, and summaries of high density oligonucleotide array probe level data. Biostatistics.

[B90] Benjamini Y, Hochberg Y (1995). Controlling the false discovery rate: a practical and powerful approach to multiple testing. Journal of the Royal Statistical Society Series B.

[B91] Rozen S, Skaletsky H (2000). Primer3 on the WWW for general users and for biologist programmers. Methods Mol Biol.

[B92] Livak KJ, Schmittgen TD (2001). Analysis of relative gene expression data using real-time quantitative PCR and the 2(-Delta Delta C(T)) method. Methods.

[B93] Vandesompele J, De Preter K, Pattyn F, Poppe B, Van Roy N, De Paepe A, Speleman F (2002). Accurate normalization of real-time quantitative RT-PCR data by geometric averaging of multiple internal control genes. Genome Biol.

[B94] Broeckling CD, Huhman DV, Farag MA, Smith JT, May GD, Mendes P, Dixon RA, Sumner LW (2005). Metabolic profiling of *Medicago truncatula *cell cultures reveals the effects of biotic and abiotic elicitors on metabolism. J Exp Bot.

[B95] Krisa S, Téguo PW, Decendit A, Deffieux G, Vercauteren J, Mérillon JM (1999). Production of ^13^C-labelled anthocyanins by *Vitis vinifera *cell suspension cultures. Phytochemistry.

[B96] Hosokawa K, Fukunaga Y, Fukushi E, Kawabata J (1995). Seven acylated anthocyanins in the blue flowers of *Hyacinthus orientalis*. Phytochem.

[B97] Deluc L, Barrieu F, Marchive C, Lauvergeat V, Decendit A, Richard T, Carde JP, Merillon JM, Hamdi S (2006). Characterization of a grapevine R2R3-MYB transcription factor that regulates the phenylpropanoid pathway. Plant Physiol.

[B98] Dai GH, Andary C, Mondolot-Cosson L, Boubals D (1995). Involvement of phenolic compounds in the resistance of grapevine callus to downy mildew (*Plasmopara viticola*). Eur J Plant Pathol.

[B99] Guralnick LJ, Ting IP (1987). Physiological changes in *Portulacaria afra *(L.) Jacq. during a summer drought and rewatering. Plant Physiol.

[B100] Priest DM, Ambrose SJ, Vaistij FE, Elias L, Higgins GS, Ross ARS, Abrams SR, Bowles DJ (2006). Use of the glucosyltransferase UGT71B6 to disturb abscisic acid homeostasis in *Arabidopsis thaliana*. Plant J.

[B101] Owen SJ, Abrams SR, Bonetta D, Cutler S (2009). Measurement of Plant Hormones by Liquid Chromatography-Mass Spectrometry. Plant Hormones: Methods and Protocols.

[B102] Oliveira C, Silva Ferreira AC, Mendes Pinto M, Hogg T, Alves F, Guedes de Pinho P (2003). Carotenoid compounds in grapes and their relationship to plant water status. J Agric Food Chem.

[B103] Osmond CB, Förster B, Pogson BJ, Allen JF, Grantt E, Golbeck JH, Osmond CB (2008). Dynamics of the truncated lutein epoxide cycle in avocado (*Persea americana *L.); implications for efficiency of light harvesting. Photosynthesis Energy from the Sun.

