# Supplementary material for: Water deficit alters differentially metabolic pathways affecting important flavor and quality traits in grape berries of Cabernet Sauvignon and Chardonnay
Source: BMC Genomics. 2009 May 8;10:212. doi: 10.1186/1471-2164-10-212 (PMC2701440; doi:10.1186/1471-2164-10-212)
Supplement: Additional file 1 — Forward and reverse primers used with qRT-PCR. This file provides a list of all of the forward and reverse primers used with qRT-PCR for the estimation of relative transcript abundance. [file 1471-2164-10-212-S1.doc]

Additional file 1. List of forward and reverse primers used in qPCR.

| **Unigene VvGi5** | **Name** | **Probesets** | **Forward** | **Reverse** |
| --- | --- | --- | --- | --- |
| TC63764 | Ferulate-5-hydroxylase | 1614502_at | CGACGTGTTCGGACTCACT | CCAAACAGTCCCTCCATGAT |
| TC54149 | Expansin-like protein | 1607674_at | GCACAGGAGCCTTGTTATCC | GCCTGTGTGTGTTCAAGGAA |
| TC63661 | Diacylglycerol kinase 2 | 1618308_at | ACTTCATTCCTCACCCCAAA | GGGAGCGGGATTAGAGAAAG |
| TC57089 | NCED1* | 1608022_at | GCAGAGGACGAGAGTGTAAAGGA | GCAGAGTAAAAACACATGAAGCTAGTG |
| AY337614 | NCED2** | - | ATGCTCAAACCGCCTCTGAT | TCCCAAGCATTCCAGAGGTG |

* Blast of these primers against Grape *Vitis vinifera* 16 K Affymetrix DNA microarrays does match to 1608022_at probeset (E-value: 2e-06; 100 % of identities) related to 9-*cis*-epoxycarotenoid dioxygenase 1.

** Blast of these primers against Grape *Vitis vinifera* 16 K Affymetrix DNA microarrays does not match to any probeset related to 9-*cis*-epoxycarotenoid dioxygenase genes.
