# Supplementary figures and images for: Water deficit alters differentially metabolic pathways affecting important flavor and quality traits in grape berries of Cabernet Sauvignon and Chardonnay
Source: BMC Genomics. 2009 May 8;10:212. doi: 10.1186/1471-2164-10-212 (PMC2701440; doi:10.1186/1471-2164-10-212)

## Slide 1
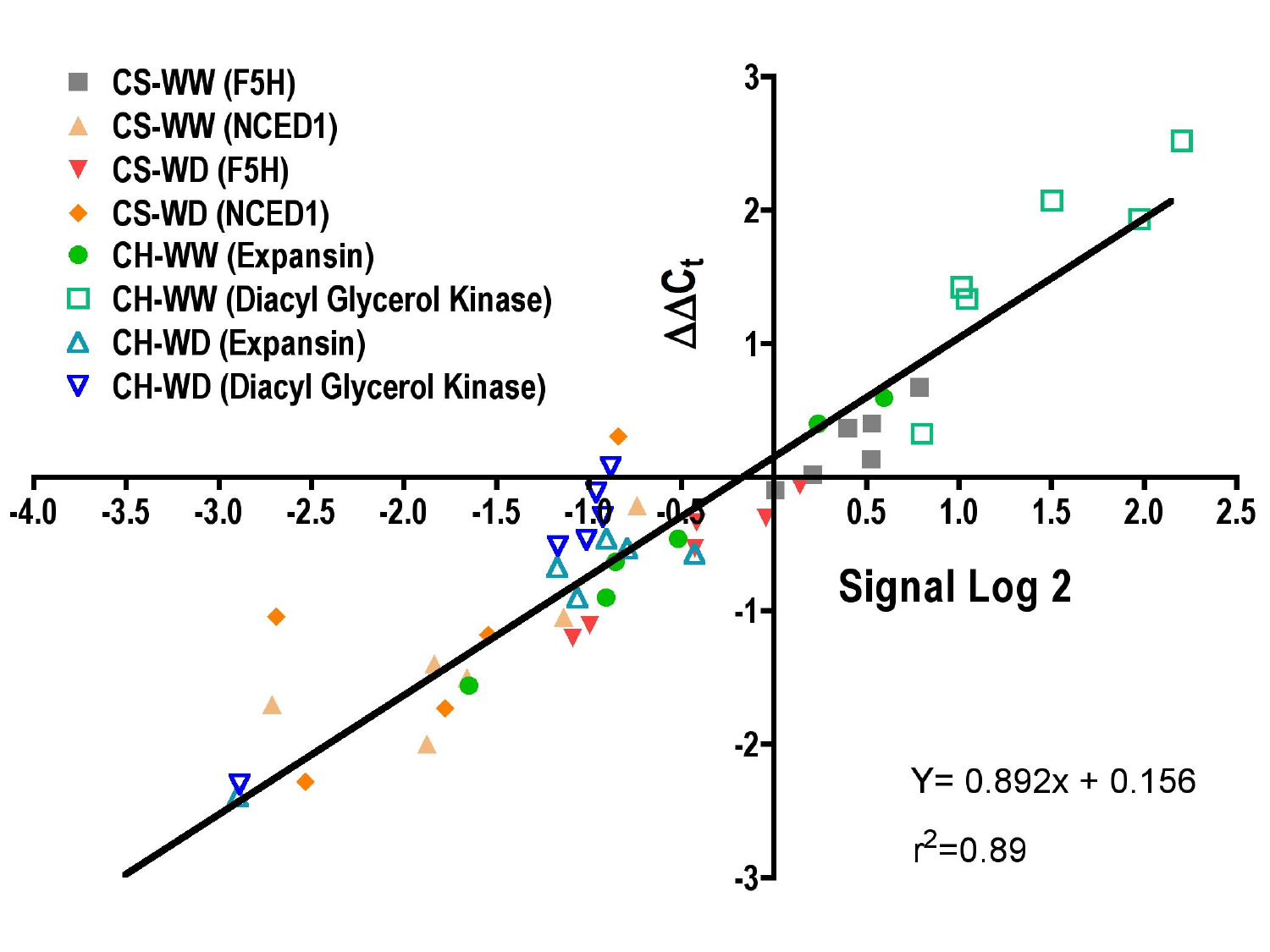

Supplement: Additional file 2 — Linear regression of the transcript abundance values of the qRT-PCR with the corresponding values of the same transcript on the microarray. qRT-PCR data are on the y-axis and the microarray data are on the x-axis. Each value represents the mean of 6 different time points for each transcript. Data were normalized to the transcript abundance of an ankyrin-repeat protein (1612584_s_at) that did not change with developmental stage or stress treatment. Ferulate-5-hydroxylase (F5H, 1614502_at, TC63764) is represented by solid grey squares (Cabernet Sauvignon with well-watered conditions; CS-WW) and by red triangles (Cabernet Sauvignon with water deficit; CS-WD). 9-cis-epoxycarotenoid dioxygenase (NCED1, 1608022_at, TC57089) is represented by orange triangles (CS-WW) and orange diamonds (CS-WD). Expansin (1607674_at, TC54149) is represented by solid green circles (Chardonnay with well-watered conditions; CH-WW) and open light blue triangles (CH-WD). Diacyl glycerol kinase 2 (1618308_at, TC63661) is represented by open green squares (CH-WW) and open dark blue triangles (CH-WD). [file 1471-2164-10-212-S2.ppt]

## Slide 1
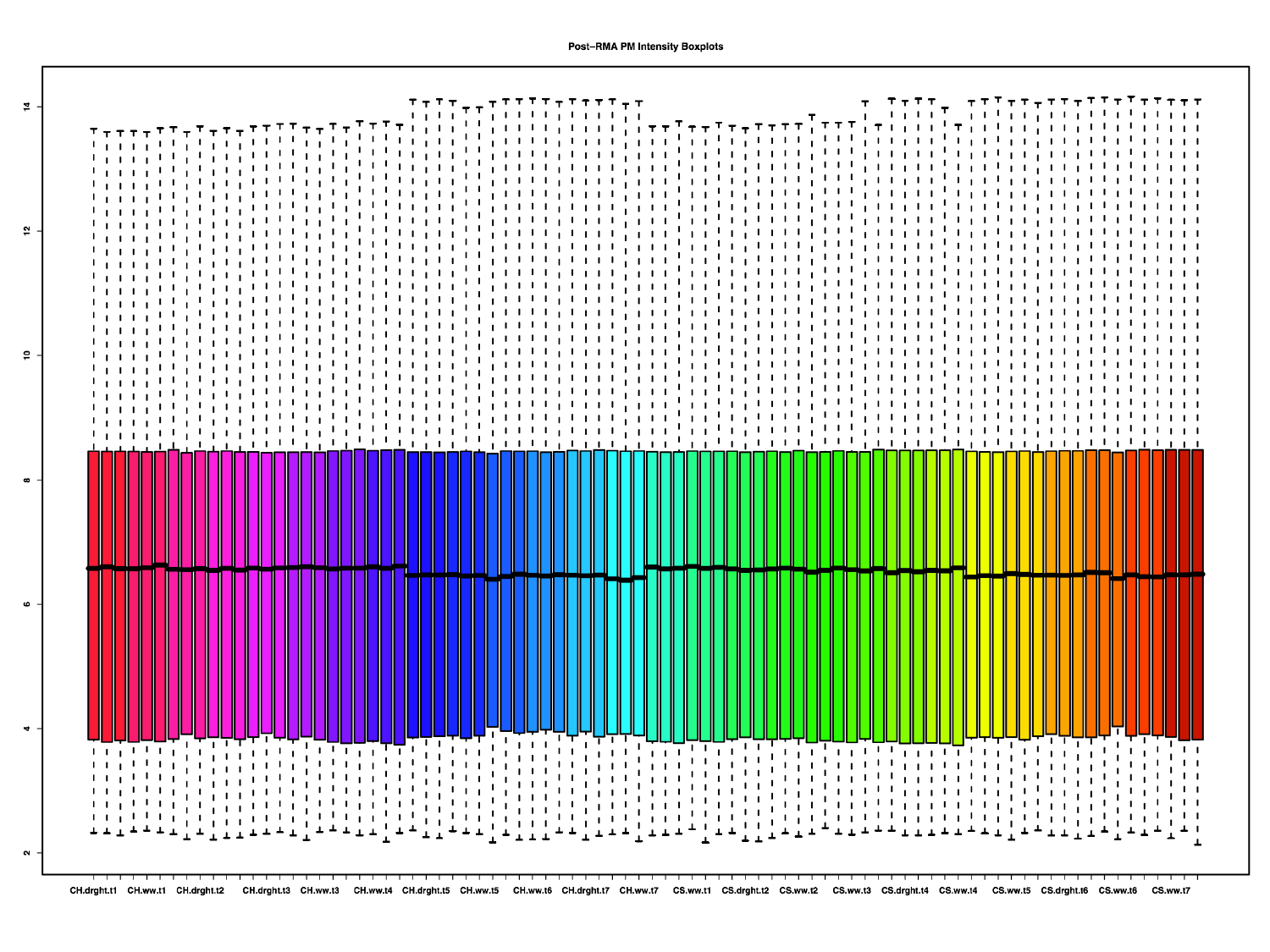

#

Supplement: Additional file 6 — Post-RMA Perfect Match (PM) intensity boxplots of all 84 Vitis Genome Arrays. This is a plot to assess the quality of the data from the arrays. [file 1471-2164-10-212-S6.ppt]

## Slide 1
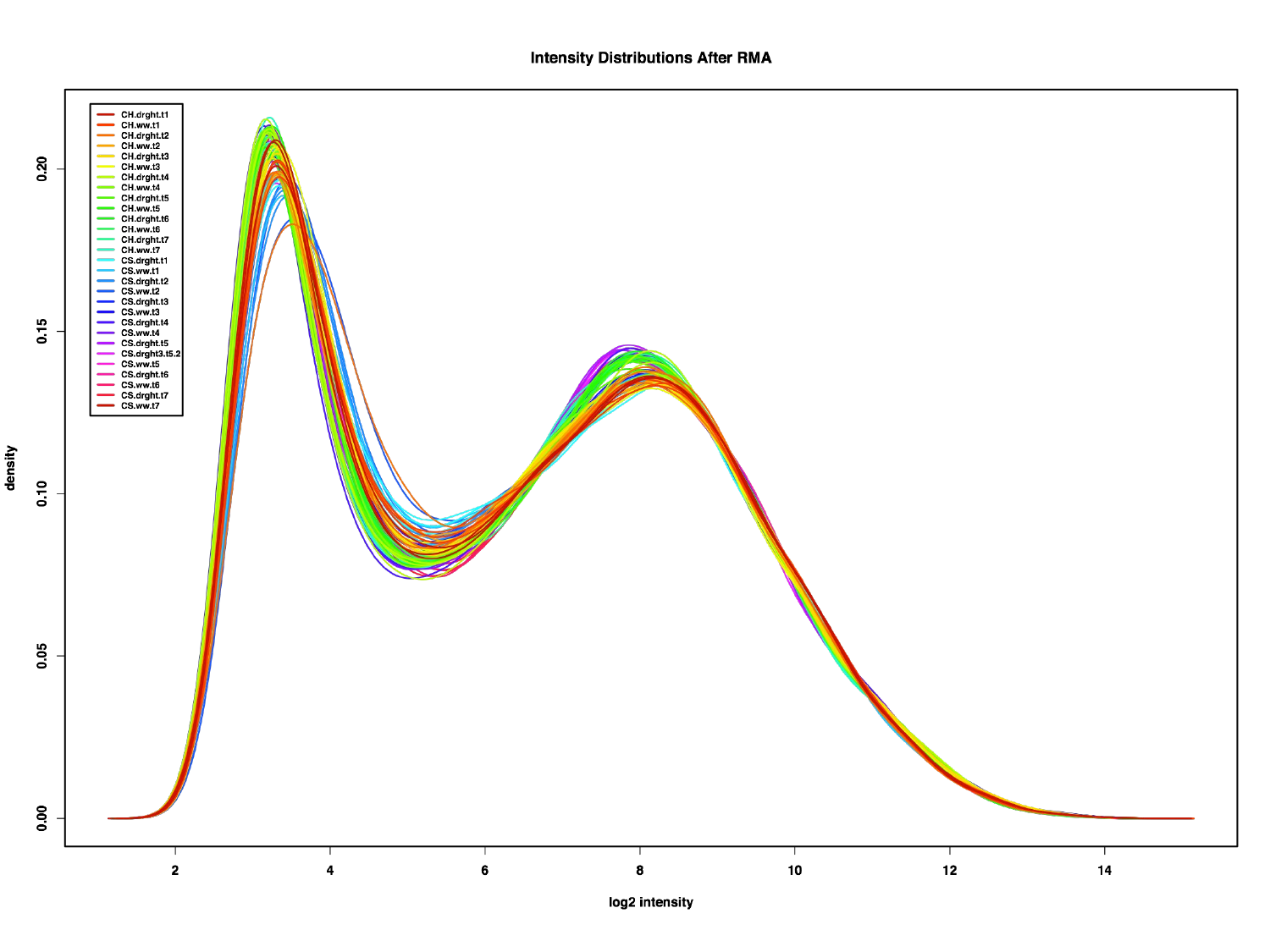

#

Supplement: Additional file 7 — Post-RMA Perfect Match (PM) intensity distributions of all 84 Vitis Genome Arrays. This is a plot to assess the quality of the data from the arrays. [file 1471-2164-10-212-S7.ppt]

## Slide 1
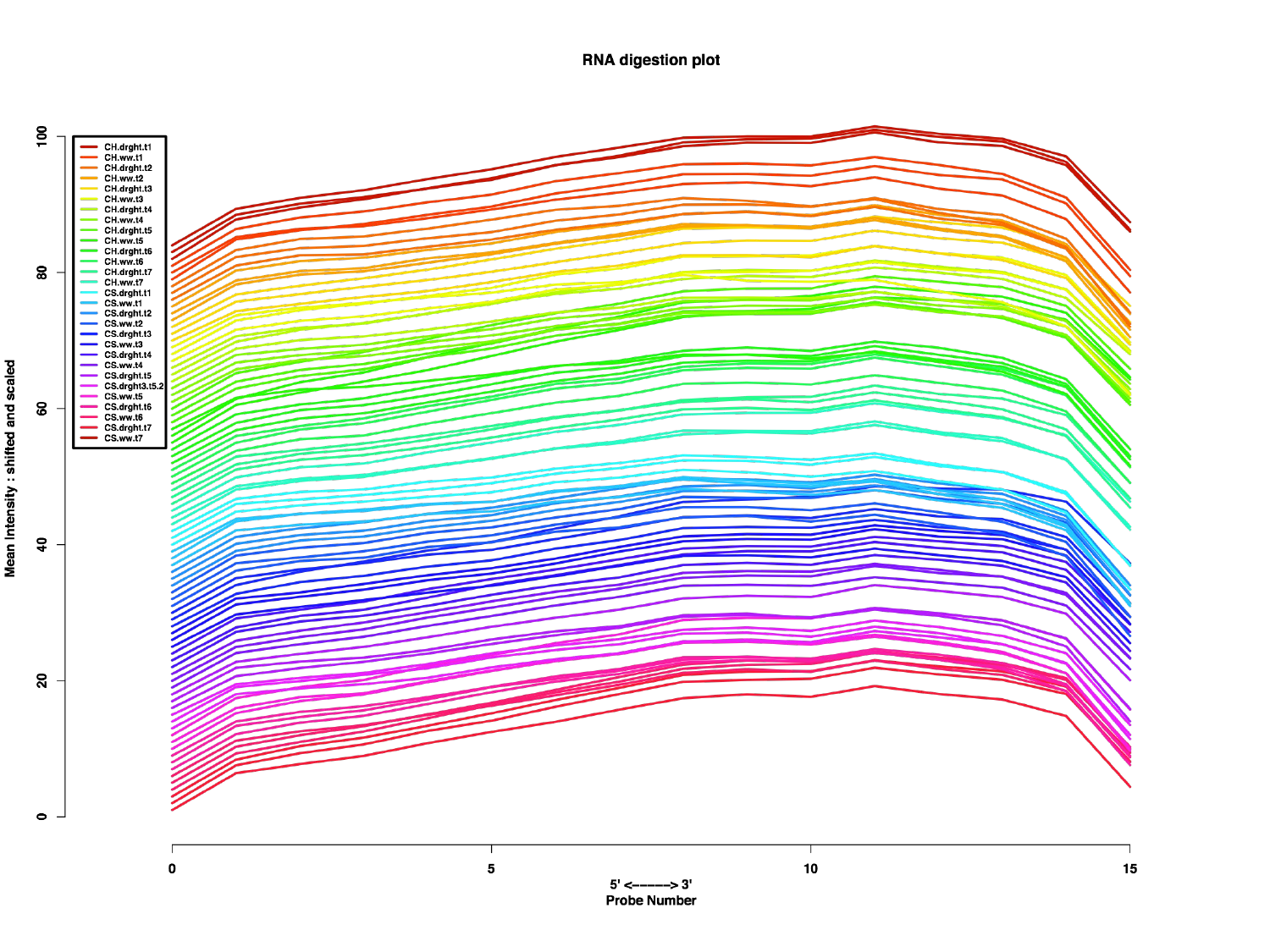

#

Supplement: Additional file 8 — RNA digestion plot of all 84 Vitis Genome Arrays. This is a plot to assess the quality of the data from the arrays. [file 1471-2164-10-212-S8.ppt]
