# Supplementary material for: Water deficit alters differentially metabolic pathways affecting important flavor and quality traits in grape berries of Cabernet Sauvignon and Chardonnay
Source: BMC Genomics. 2009 May 8;10:212. doi: 10.1186/1471-2164-10-212 (PMC2701440; doi:10.1186/1471-2164-10-212)
Supplement: Additional file 9 — A representative HPLC chromatogram for carotenoid identification and quantification. This is a plot to assess the quality of the data and the retention times from the chromatogram. [file 1471-2164-10-212-S9.ppt]

## Slide 1
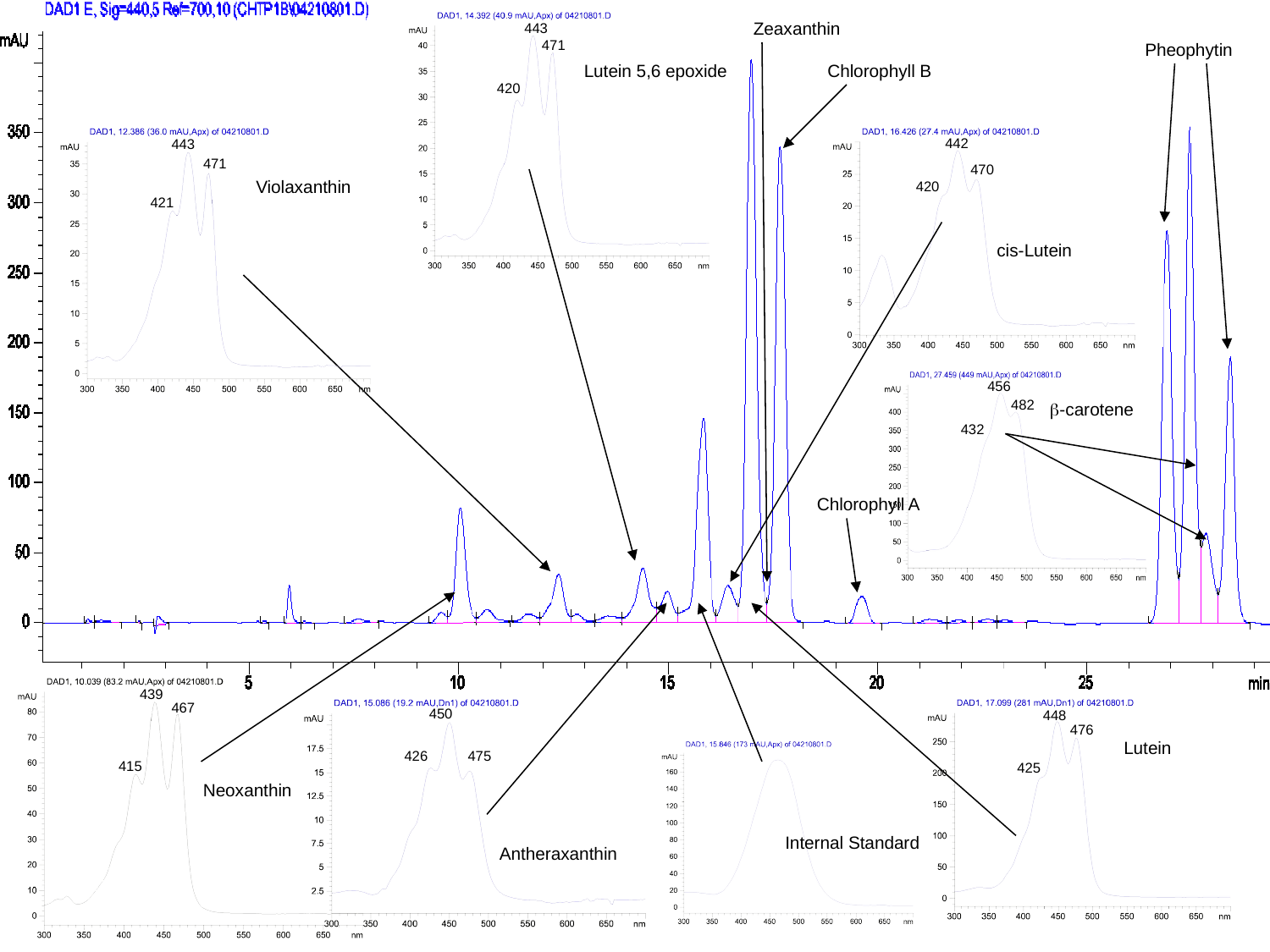

Zeaxanthin
443
471
Pheophytin
Lutein 5,6 epoxide
Chlorophyll B
420
442
443
471
470
Violaxanthin
420
421
cis-Lutein
456
482
β-carotene
432
Chlorophyll A
439
467
450
448
476
Lutein
426
475
415
425
Neoxanthin
Internal Standard
Antheraxanthin
